# Early and progressive retinal microglial changes in APP^NL-F/NL-F^ mouse model of Alzheimer’s disease revealed by an automated image analysis software

**DOI:** 10.3389/fnagi.2025.1712480

**Published:** 2026-01-15

**Authors:** Lidia Sánchez-Puebla, Inés López-Cuenca, Miguel A. Sánchez-Puebla, Ana Granados, Ana I. Ramírez, Juan Llorens, Takaomi C. Saido, Takashi Saito, Carmen Nieto-Vaquero, María A. Moro, Valentín Moreno, José M. Ramírez, Rosa de Hoz

**Affiliations:** 1 Ramon Castroviejo Institute for Ophthalmic Research, Complutense University of Madrid, Madrid, Spain; 2 Health Research Institute of the Hospital Clínico San Carlos (IdISSC), Madrid, Spain; 3 Department of Immunology, Ophthalmology and ENT, School of Medicine, Complutense University of Madrid, Madrid, Spain; 4 Department of Immunology, Ophthalmology and ENT, Faculty of Optics and Optometry, Complutense University of Madrid, Madrid, Spain; 5 Department of Computer Science, Carlos III University of Madrid, Leganés, Spain; 6 Laboratory for Proteolytic Neuroscience, Brain Science Institute, RIKEN, Wako, Japan; 7 Faculty of Medical Sciences, Institute of Brain Science, Nagoya City University, Nagoya, Japan; 8 Neurovascular Pathophysiology, Cardiovascular Risk Factor and Brain Function Programme, Centro Nacional de Investigaciones Cardiovasculares (CNIC), Madrid, Spain; 9 Hospital 12 de Octubre Research Institute (i+12), Madrid, Spain; 10 University Institute for Research in Neurochemistry, Complutense University of Madrid, Madrid, Spain

**Keywords:** Alzheimer’s disease, APP^NL-F/NL-F^ mouse model, automated image analysis, morphological quantification, neuroinflammation, retinal microglia

## Abstract

Alzheimer’s disease (AD) is characterized by the accumulation of misfolded proteins that trigger neuroinflammation and neuronal loss. The retina, as an extension of the central nervous system, mirrors these pathological processes and represents a potential biomarker. Microglial activation, a key component of neuroinflammation, can be morphologically assessed through automated image analysis. This study performed a quantitative and morphological analysis of retinal microglia in the APP^NL-F/NL-F^ mouse model of AD across aging (6–20 months) and comparing them with age-matched C57BL/6 J controls using an automated image analysis software. A cross-sectional design was applied to 72 mice (36 APP^NL-F/NL-F^ and 36 WT). Retinas samples were processed by Iba-1 immunohistochemistry. Quantified parameters included cell number, soma size, arborization area, skeletonization, fluorescence intensity, and Feret’s Diameter Ratio across OS, OPL, IPL, and NFL/GCL layers. Image analysis was performed using a custom automated system, called MorphoSomas, specifically developed for the comprehensive morphological assessment of microglia. Age-dependent changes were observed in both groups. WT mice showed a later and more gradual activation pattern, whereas APP^NL-F/NL-F^ mice exhibited early activation from 6 months, characterized by increased cell number and soma size, followed by reductions in arborization and skeletonization, indicating progressive activation. The automated system allowed precise and reproducible assessment, highlighting significant differences between groups and retinal layers. In conclusion, retinal microglia in APP^NL-F/NL-F^ mice exhibit early and biphasic activation followed by signs of dysfunction, reflecting AD neuropathology. Automated analysis enhances objectivity and efficiency in morphological studies. These findings support the retina as a promising, non-invasive biomarker for early AD detection.

## 1 Introduction

Alzheimer’s disease (AD) is the leading cause of dementia worldwide (Tahami Monfared et al., 2022). This condition is marked by disruptions in the metabolism and clearance of beta-amyloid (Aβ) and tau proteins, which result in the accumulation of Aβ plaques and neurofibrillary tangles within the central nervous system (CNS) (Hardy and Higgings, 1992; Gulisano et al., 2018). Misfolded proteins associated with AD are known to contribute to the development of tauopathies and inflammatory responses, ultimately causing significant synaptic and neuronal loss in the brain (De Strooper and Karran, 2016; Blanks et al., 1996).

Inflammation is a fundamental defensive mechanism of the body in response to injury, aimed at preserving and restoring tissue integrity. Within the CNS, neuroinflammation plays a critical role in safeguarding neural tissue. However, when this inflammatory response becomes chronic or dysregulated, it may lead to detrimental effects, including cellular damage. In this context, glial cells—particularly astrocytes and microglia—are recognized as fundamental contributors to the neuroinflammatory process associated with various neurodegenerative disorders (Cherry et al., 2014). In this process microglial activation play a key role. Microglial activation exists along a dynamic continuum rather than distinct M1/M2 polarization states, with functional outcomes ranging from neurotoxic to neuroprotective depending on context and signalling cues. This functional diversity suggests that microglial activation may play a pivotal role in controlling neuroinflammatory responses (Glass et al., 2010). The activation of microglia is modulated by interactions with various cell types, including neurons, astrocytes, and T lymphocytes. When neuroinflammation is initiated by the accumulation of misfolded proteins such as amyloid-*β* (Aβ), or phosphorylated tau (pTau), peripheral immune cells can infiltrate the CNS, further stimulate resident microglia and amplify the inflammatory response (Ramirez et al., 2017).

Neuroinflammatory responses are not limited to the brain but also manifest in the retina, given its anatomical and developmental continuity with the CNS. Monitoring inflammatory activity in retinal tissue may offer a valuable, non-invasive approach for the early detection and progression tracking of neurodegenerative disorders. Multiple studies have shown that the retina also undergoes changes related to AD, both in humans (Zheng et al., 2023; Salobrar-García et al., 2019; Alber et al., 2020) and in transgenic mouse models (Salobrar-García et al., 2021; Grimaldi et al., 2018; Guo et al., 2021; Gupta et al., 2016; Edwards et al., 2014). These models have helped establish correlations between retinal and cerebral abnormalities (Philipson et al., 2010; Goncalves and Antonetti, 2022).

One such model is the APP^NL-F/NL-F^ mouse, which exhibits increased production of Aβ42, promoting the formation of pathological Aβ deposits in the cerebral cortex and hippocampus. This accumulation triggers the activation and infiltration of microglia and astrocytes around the Aβ plaques starting at 6 months of age (Saito and Saido, 2018). The model replicates several pathological features observed in AD patients, making it a valuable tool for investigating the role of amyloidosis in neuroinflammatory processes (Sasaguri et al., 2017). Furthermore, early retinal structural and vascular alterations that precede cognitive symptoms make this model more representative of human AD than other transgenic models. Between 6 and 20 months of age, optical coherence tomography (OCT) has revealed changes in retinal layer thickness, including both thinning and thickening of the total retina, as well as its inner and outer layers of retina. The thinning of inner retinal layers observed at 6, 12, and 15 months may reflect ongoing neurodegenerative processes. In contrast, the thickening of outer retinal layers, particularly noted at 6 and 17 months, could suggest a neuroinflammatory response, either primary or associated with *β*-amyloid accumulation (Sánchez-Puebla et al., 2024a).

To the best of our knowledge, no previous studies have employed an automated image analysis software specifically developed to detect, quantify, and characterize microglial somas, perform skeletonization, and accurately measure the arborization area of these cells in retinal tissue from the APP^NL-F/NL-F^ mouse model. Given the advantages of this experimental model, the aim of the present study was to conduct a quantitative and morphological assessment of retinal microglial cells over time (at 6, 9, 12, 15, 17, and 20 months of age), using an innovative analytical tool, and to compare these findings with age-matched wild-type controls in a well-validated model of Alzheimer’s disease.

## 2 Methods

The experimental procedures were conducted using male APP^NL-F/NL-F^ mice, which were genetically engineered through a knock-in approach to introduce the Swedish (KM670/671NL) and Beyreuther/Iberian (I716F) mutations into the endogenous APP gene, as previously described by Saito et al. (2014). Age-matched wild-type (WT) mice of the C57BL/6 J strain were used as controls. Male mice were selected to minimise biological variability associated with hormonal fluctuations, as females have been reported to exhibit a higher pathological burden and enhanced neuroinflammatory responses, potentially linked to sex-specific hormonal regulation (Carroll et al., 2010; Mielke et al., 2014).

In this model, the Aβ domain of the murine APP gene has been replaced with its human counterpart. The Swedish mutation (KM670/671NL) leads to an overall increase in Aβ40 and Aβ42 production, while the Beyreuther/Iberian mutation (I716F) specifically elevates the Aβ42/Aβ40 ratio (Saito et al., 2014). A key strength of this model lies in the use of knock-in technology to introduce these humanized mutations directly into the endogenous APP locus, preserving physiological expression patterns (Saito et al., 2014). To enhance the development of amyloid pathology and eliminate endogenous murine Aβ, the mice are maintained in a homozygous state (Sasaguri et al., 2017). Consequently, control animals are not littermates. However, this is unlikely to introduce significant variability, as the mutant line has been backcrossed onto a pure C57BL/6 J background for over ten generations.

### 2.1 Experimental groups

A cross-sectional case–control study was conducted using mice at 6, 9, 12, 15, 17, and 20 months of age. The study included two groups: an experimental group consisting of APP^NL-F/NL-F^ transgenic mice (*n* = 36) and a control group of C57BL/6 J wild-type mice (*n* = 36). At each time point, six mice from each group were analysed, ensuring balanced comparisons across all ages evaluated. Only the left eyes of the animals were included in the present analyses, while the right eyes were preserved for future studies involving complementary retinal analyses.

All animals were housed under standardized environmental conditions, including a 12-h light/dark cycle with ambient light levels ranging from 9 to 24 lux, and controlled temperature. Food and water were provided ad libitum. The study was carried out at the Faculty of Medicine, Complutense University of Madrid.

All experimental procedures complied with Directive 2010/63/EU of the European Parliament and Spanish legislation (Real Decreto 53/2013) governing the protection of animals used for scientific purposes. Ethical approval was granted by the Animal Welfare Committee of the Complutense University (PROEX No. 047/16). The study adhered to the ARVO Statement for the Use of Animals in Ophthalmic and Vision Research, and all efforts were made to minimize animal use and suffering.

### 2.2 Immunohistochemistry

Following intraperitoneal anesthesia, animals underwent transcardial perfusion with 0.9% saline solution (NaCl), followed by 4% paraformaldehyde (PFA) in 0.1 M phosphate buffer, both maintained at 4 °C. Postmortem eyes were enucleated, and a small incision was made in the anterior chamber at the limbus to enhance fixation. To preserve ocular orientation, a suture was placed at the posterior pole of the eye, and additional anatomical landmarks such as the nasal caruncle and rectus muscles were used for reference (de Hoz et al., 2018). The eyes were then immersed in 4% PFA at 4 °C overnight. The next day, they were rinsed three times for 15 min each in phosphate-buffered saline (PBS, pH 7.4). For whole mounts, the cornea and lens were removed, and the retina was carefully dissected from the choroid using a fine brush. The retinas underwent cryoprotection by immersion in sucrose solutions with progressively increasing concentrations (10, 20, and 30%) for 1 h, 2 h, and overnight, respectively, at 4 °C. Afterwards, the tissues were frozen using liquid nitrogen and stored at −80 °C until further use.

For microglial analysis, these whole-mounted retinas were subjected to immunohistochemical staining following established methods (Ramírez et al., 2020). After PBS washes, whole-mount retinas were incubated in a blocking solution containing 2% Triton X-100 and 10% Animal-Free Blocker (SP-5035; Vector Laboratories, CA, USA) in 0.1 M PBS. Samples were then incubated with a rabbit anti-Iba-1 antibody (Wako, Osaka, Japan) diluted 1:600 in a solution of 1% Animal-Free Blocker, 2% Triton X-100, and PBS, incubation was performed for 3 days. Following primary antibody incubation, samples were washed three times in PBS and incubated with donkey anti-rabbit IgG conjugated to Alexa Fluor 594 (Invitrogen, Paisley, UK), diluted 1:800 in 0.1 M PBS for 2 days. After final PBS washes, whole-mounts were mounted with VECTASHIELD Vibrance® (H-1700, Vector Laboratories, Inc., Canadá). Two types of negative controls were included. In the first, the primary antibody was omitted, and tissues were incubated with only the secondary antibody and diluent. In the second control, tissues were exposed solely to the diluents used for both primary and secondary antibodies. This latter control was used to evaluate the contribution of tissue autofluorescence to the observed signal (Triviño et al., 2002).

Immunostained samples were analysed using a Zeiss Axio Imager M.2 fluorescence microscope (Carl Zeiss AG, Oberkochen, Germany), equipped with an Axio Cam 503 Mono high-resolution camera and an Apotome-2 module. The system included filter sets for Alexa Fluor 488 (Zeiss 10) and Alexa Fluor 594 (Zeiss 64). Image acquisition and analysis were performed using ZEN2 software (Carl Zeiss), ensuring consistent illumination and magnification settings. Whole-mounts were analysed in three dimensions (XYZ axes) using a motorized stage, enabling precise spatial localization of microglial structures. Elements located within the same x-z plane were considered to reside in the same focal plane. Image acquisition was performed using the ApoTome system, which improves axial resolution by removing out-of-focus light and generating high-contrast optical sections. Z-stack images were captured along the Z-axis using a 20 × objective and processed using Axiovision software (version 4.2, Carl Zeiss). Final figure panels were assembled using Adobe Photoshop CS4 Extended (version 10.0, Adobe Systems, San José, CA, USA).

**Figure 1:**
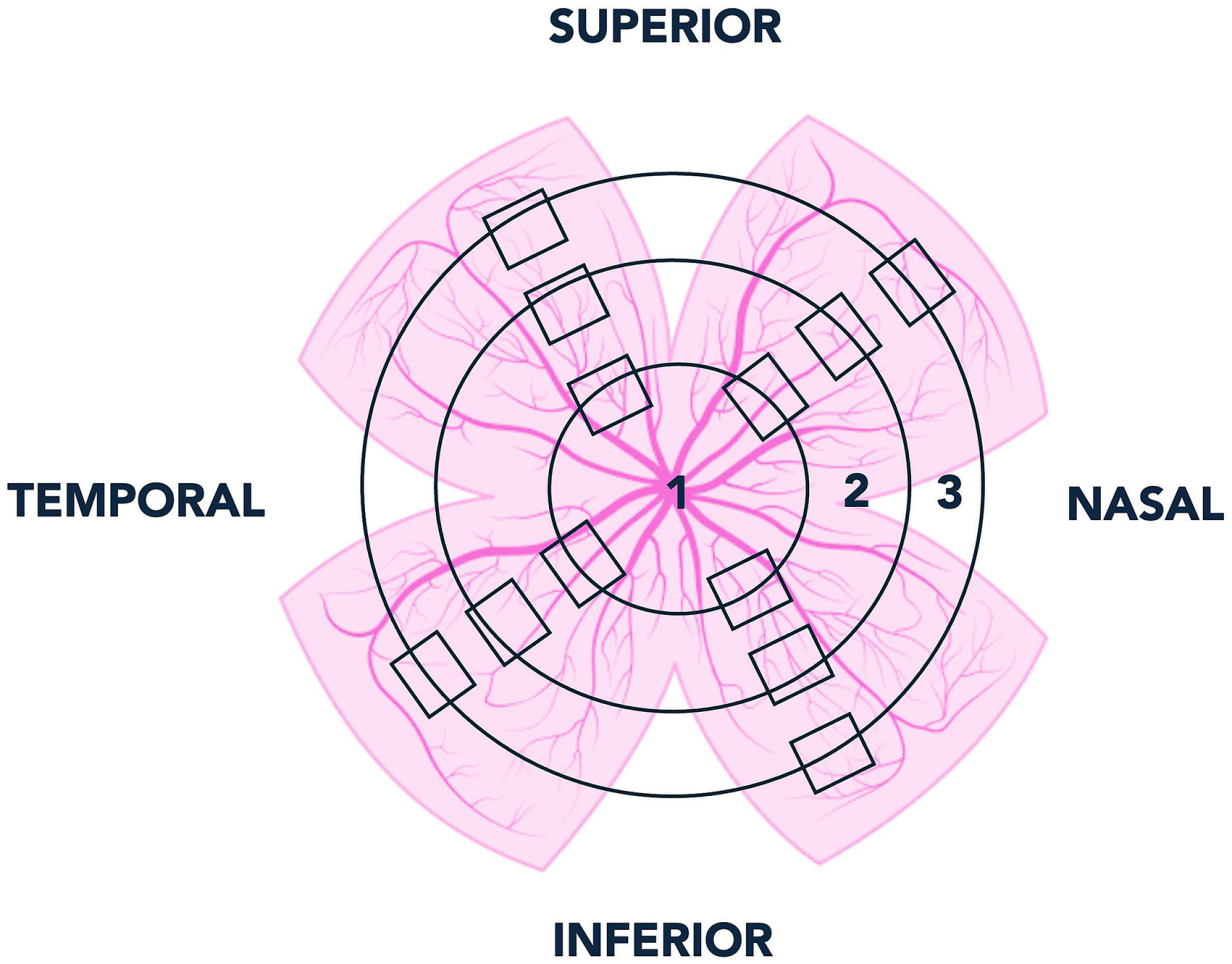
Schematic representation of the systematic sampling strategy used for retinal whole mounts. The retina was divided into nasal, temporal, superior, and inferior quadrants. In each quadrant, three fields were imaged at increasing radial distances from the optic disc (zones 1, 2, and 3), covering both meridians (*X* and *Y* axes).

To ensure systematic sampling, each retinal whole-mount was scanned using the microscope’s automated stage, covering both horizontal and vertical meridians (*X* and *Y* axes). The retina was divided into four quadrants (nasal, temporal, superior, and inferior). In each quadrant, three images were acquired at increasing radial distances from the optic disc toward the periphery (proximal, intermediate, and peripheral regions), as illustrated in Figure 1. Each field of view analysed corresponded to an area of 0.1502 mm^2^, resulting in twelve microphotographs per retina (four quadrants × three eccentricities). Thus, for each experimental time point, twelve images were obtained per retina, and since six retinas were analysed per group, a total of seventy-two images were evaluated for each group and time point. Therefore, one hundred forty-four images were analysed at each experimental age (WT and APP groups combined).

These images focused on retinal layers where microglial cells were observed: the outer segments of the photoreceptors (OS), the outer plexiform layer (OPL), the inner plexiform layer (IPL), and the nerve fiber layer combined with the ganglion cell layer (NFL/GCL).

**Figure 2:**
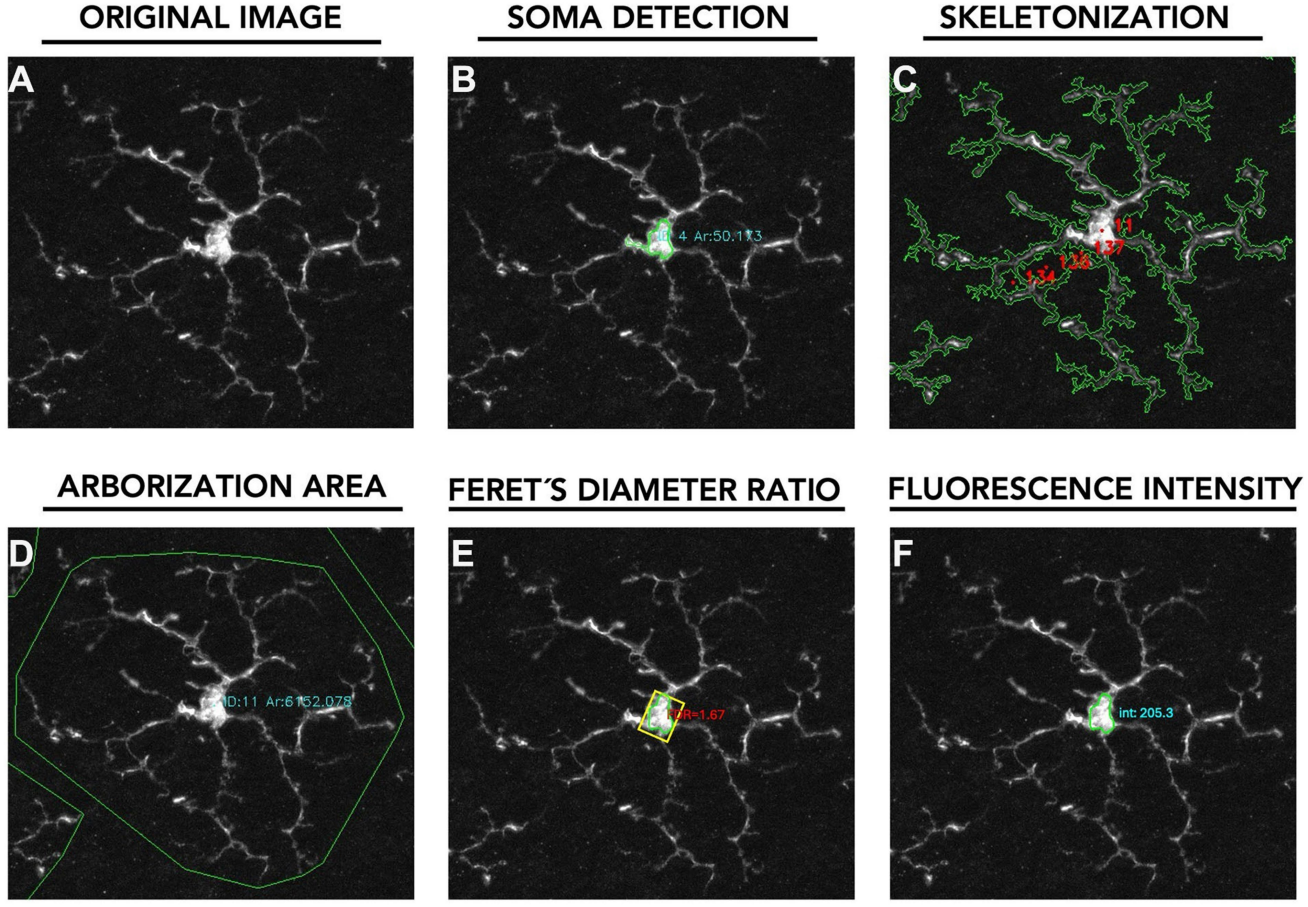
Pipeline for quantitative analysis of microglial morphology and fluorescence intensity. **(A)** Original image: showing a fluorescently labelled microglial cell. **(B)** Soma detection with area measurement. **(C)** Skeletonization of microglial processes for structural analysis. **(D)** Arborization area defined by the convex hull enclosing the cell’s processes. **(E)** Feret’s diameter ratio calculation for morphological quantification. **(F)** Fluorescence intensity measurement within the soma region.

Images were acquired at 2 μm intervals along the Z-axis, enabling detailed analysis of the following variables within the specified layers (Figure 2):

1. Total number of Iba-1 + microglial cells in the OS, OPL, IPL, and NFL/GCL.
2. Soma size of microglial cells in the OPL and IPL.
3. Skeletonization area of microglial processes in the OPL and IPL.
4. Arborization area of microglial cells in the OPL and IPL.
5. Feret’s diameter Ratio in IPL and OPL.
6. Fluorescence intensity of Iba-1 staining in IPL and OPL.

All images acquired were used for the quantitative representation of microglial cell number. Morphometric parameters, including soma area, arborization area, skeletonization area, Feret’s diameter ratio, and fluorescence intensity, were obtained individually for each microglial cell identified within the analysed fields. Systematic imaging was performed for the OPL and the IPL, where microglial cells were consistently present. In contrast, in the OS and NFL/GCL layers, microglial cells were not consistently detected in all retinas. Therefore, no systematic imaging was carried out in these layers; instead, the total number of Iba-1^+^ cells was manually counted whenever cells were present.

### 2.3 Automated image analysis system for retinal microglia

An automated software tool was developed in collaboration with Carlos III University of Madrid (UC3M) to perform morphological analysis of Iba-1^+^ microglial cells in the retina. This system, called *MorphoSomas*, integrates advanced image processing algorithms with expert validation tools to accurately detect cell somas, generate skeletonized structures, and quantify arborization areas. The detailed procedures for soma segmentation, skeletonization, and arborization measurement have been previously described in (Sánchez-Puebla et al., 2025), where the methodology was validated and its automated implementation established.

In the current study, this analysis pipeline was extended to include two additional quantitative parameters: Feret’s Diameter Ratio (FDR) and soma fluorescence intensity.

The FDR is a shape descriptor widely used to evaluate cellular elongation and orientation. It is computed as the ratio between the maximum and minimum Feret’s diameters, which represent the longest and shortest distances between any two parallel tangents to the soma contour, respectively:


$${\text{Feret}}^{'}s\;\;\text{Diameter Ratio}=\frac{\text{Fmax}}{\text{Fmin}}$$

By definition, FDR values are ≥1. A perfectly circular soma yields an FDR of 1, whereas higher values indicate elongation, which may reflect microglial activation or morphological transition states. In this study, FDR was specifically calculated in the OPL and IPL, where microglial density is highest. Other authors have reported that ramified microglia typically exhibit FDR values <3, whereas more activated or amoeboid morphologies, including rod-shaped cells, often display FDR ≥ 3. However, it is important to note that this measure exclusively evaluates soma shape and should not be interpreted as a direct indicator of cellular ramification, which requires additional parameters such as skeletonization and arborization analysis (Choi et al., 2022).

Additionally, the software quantifies soma fluorescence intensity of Iba-1 + by measuring the mean grayscale value of all pixels within the segmented soma area. Pixel intensity values range from 0 (black) to 255 (white). Our tool includes a custom-built automatic correction algorithm that normalizes intensity measurements across images. This feature compensates for technical variability in acquisition conditions such as exposure time, excitation strength, background signal, or interference from neighbouring cells. For result representation, the mean intensity values were used and interpreted using the RGB scale available in the Paint software (11.2401.20.0 version).

### 2.4 Statistical analysis

Temporal progression within each group was assessed using one-way ANOVA followed by Tukey’s *post hoc* correction for multiple comparisons. For case–control comparisons between groups (WT vs. APP^NL-F/NL-F^) at each time point, the non-parametric Mann–Whitney U test was used. Statistical significance was defined as follows: *p* < 0.05 (*), *p* < 0.01 (**), *p* < 0.001 (***), and *p* < 0.0001 (****). Statistical analyses were performed using GraphPad Prism software, version 9.4.1 (GraphPad Software, La Jolla, CA, USA).

## 3 Results

**Figure 3:**
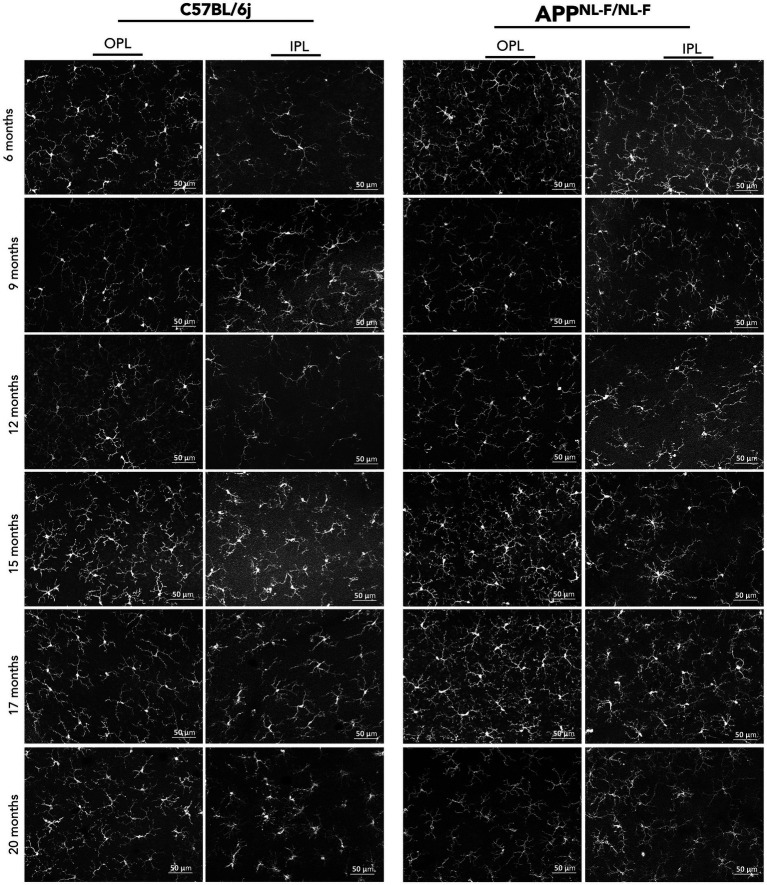
Age-dependent changes in microglial morphology in the retina of C57BL/6J and APP^NL-F/NL-F^ mice. Representative confocal images of Iba-1^+^ microglial cells in the outer plexiform layer (OPL) and inner plexiform layer (IPL) of the retina from C57BL/6J (left) and APP^NL-F/NL-F^ mice (right) at 6, 9, 12, 15, 17, and 20 months of age. Progressive morphological alterations can be observed across aging, particularly in the APP^NL-F/NL-F^ group.

In C57BL/6J mice, microglial cells maintained a relatively stable morphology across time, characterized by highly ramified processes and a regular distribution in both OPL and IPL layers. Subtle changes in arborization complexity and process thickness were observed with aging, particularly beyond 15 months, but overall morphology remained consistent (Figure 3).

In contrast, microglia from APP^NL-F/NL-F^ mice displayed progressive and marked morphological alterations with age. As early as 12 months, microglial processes appeared less ramified and more hypertrophic in both layers. These changes became more pronounced at 15, 17, and 20 months, with evident soma enlargement, retraction of processes, and clustering of microglial cells—particularly in the IPL. These features are indicative of a reactive or activated microglial phenotype and suggest a neuroinflammatory response associated with amyloid pathology in the APP^NL-F/NL-F^ (Figure 3).

All these morphological alterations will be described in detail throughout the results section, including quantitative analyses of soma size, skeletonization area, arborization area, FDR and fluorescence intensity.

### 3.1 Temporal study of the APP^NL-F/NL-F^ model

#### 3.1.1 Number of Iba-1+ cells

In APP^NL-F/NL-F^ retinas, the number of Iba-1^+^ microglial cells per mm^2^ showed a significant and progressive increase with age across all analysed retinal layers.

##### 3.1.1.1 Outer segment layer (OS)

**Figure 4:**
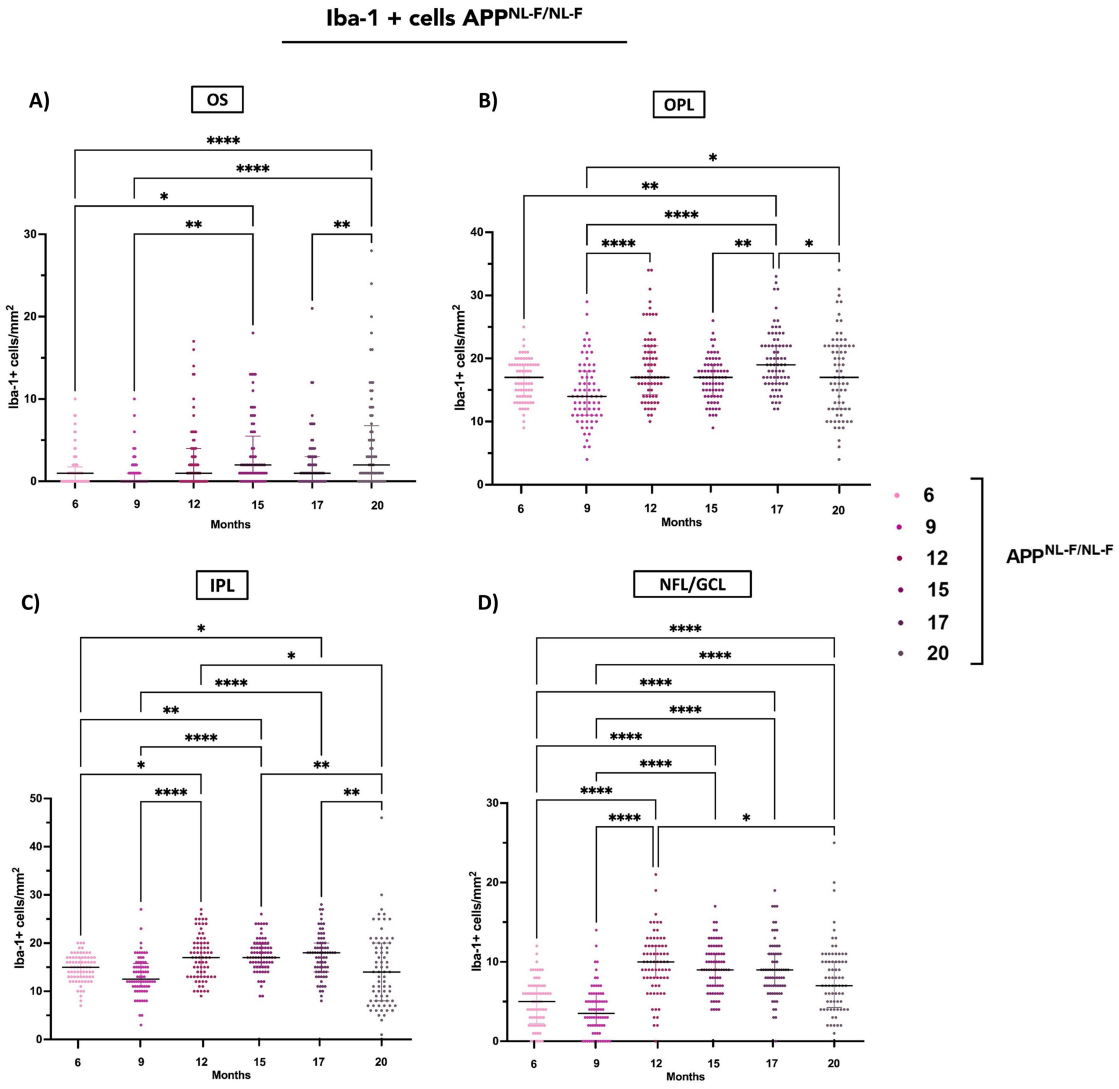
Quantification of Iba-1^+^ cells in different retinal layers (OS, OPL, IPL, NFL/GCL) across age groups in APP^NLF-NLF^ group. **(A)** Outer segment (OS), **(B)** outer plexiform layer (OPL), **(C)** inner plexiform layer (IPL), and **(D)** nerve fiber layer/ganglion cell layer (NFL–GCL). Data are shown as median with interquartile range. Each data point represents the total number of Iba-1^+^ microglial cells identified within a systematically defined anatomical field of 0.1502 mm^2^, in which all microglial cells present were counted. Statistical significance is shown as *p* < 0.05 (*), *p*< 0.01 (**), *p* < 0.001 (***), and *p*< 0.0001(****).

In the OS, a highly significant increase in Iba-1^+^ microglial cell density was observed between 6 and 20 months, as well as between 6 and 15 months. Additionally, 9-month values were significantly lower than those at 15 and 20 months. Further increases were also detected between 17 and 20 months (Figure 4A and Supplementary Table S1).

##### 3.1.1.2 Outer plexiform layer (OPL)

In the OPL, significant increases in microglial cells density were found between 6 and 17 months, and between 9 and both 12 and 17 months. A moderate significant increase was also noted between 9 and 20 months. Additional significant increases were found between 15 and 17 months, while a significant reduction was observed from 17 to 20 months (Figures 3, 4B and Supplementary Table S2).

##### 3.1.1.3 Inner plexiform layer (IPL)

In the IPL, a significant increase in Iba-1^+^ cell density was observed between 6 and 12 months, 6 and 15 months, and 6 and 17 months. Comparisons between 9 months and 12, 15, and 17 months revealed highly significant increases. However, a decline was observed at 20 months when compared to 12, 15, and 17 months a possible change in the later stages like in OPL (Figures 3, 4C and Supplementary Table S3).

##### 3.1.1.4 Nerve fiber layer/ ganglion cell layer (NFL/GC)

A progressive increase in Iba-1^+^ cell density was evident over time. In comparison to 6 months, we observed number cells values significantly elevated at 12, 15,17, and 20. Additionally, also we found an increase of number cells at 9 months related to 6 months. While a significant reduction was only detected between 12 and 20 months. Pairwise comparisons further confirmed significant increase between 9 months and 12, 15, and 17 months, highlighting a sustained microglial response throughout aging (Figure 4D and Supplementary Table S4).

#### 3.1.2 Soma size

##### 3.1.2.1 Outer plexiform layer (OPL)

Soma size in the OPL of APP^NL-F/NL-F^ mice exhibited significant temporal fluctuations. Notable increases were observed when comparing 6 months to 9, 17, and 20 months, indicating early and sustained morphological changes. Interestingly, a reduction was detected between 6 and 12 months, suggesting a transient decrease before subsequent enlargement.

**Figure 5:**
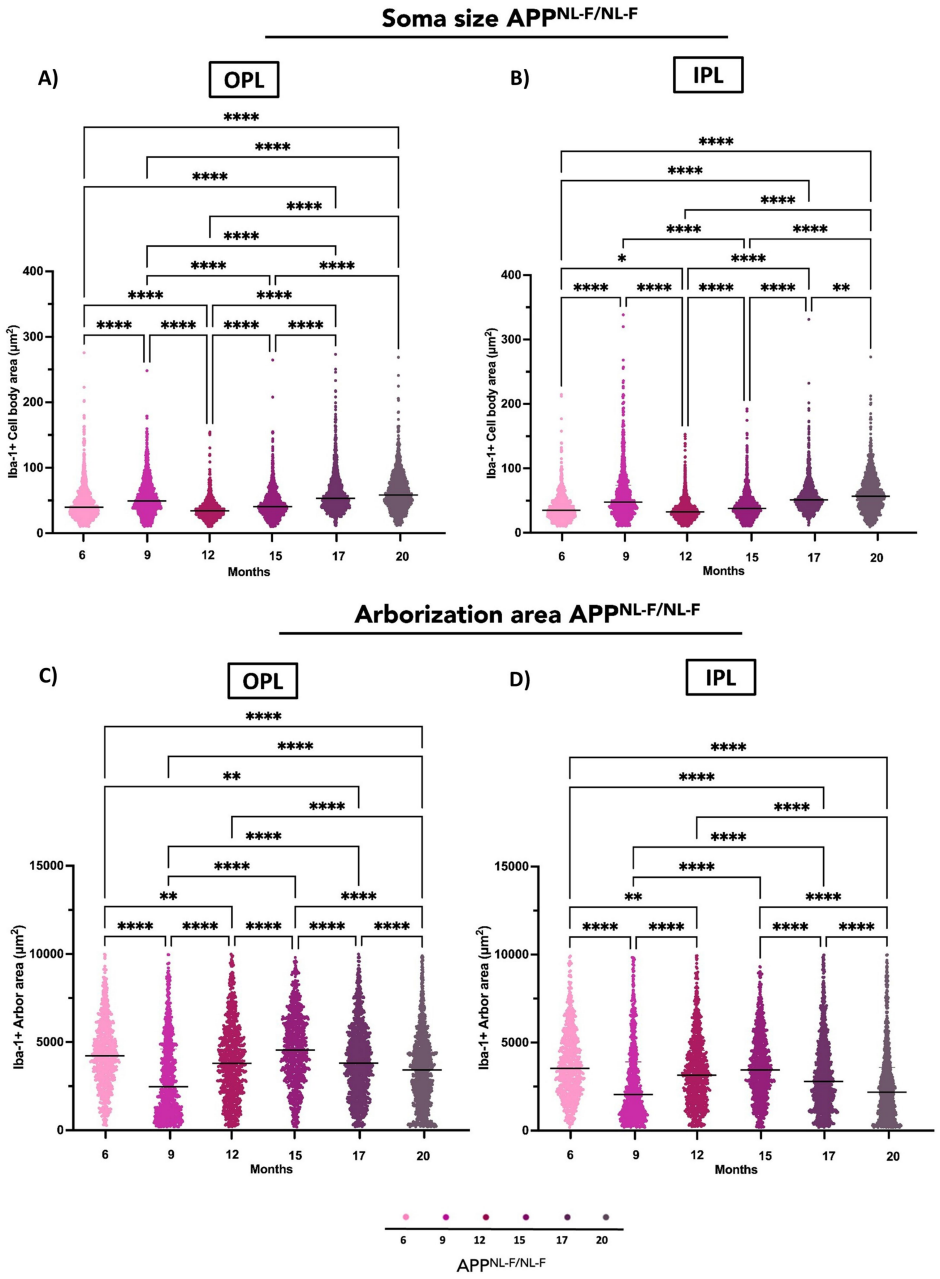
Quantitative analysis of Iba-1^+^ microglial morphology in the retina of *APP^NL-F/NL-F^* mice at different ages (6, 9, 12, 15, 17, and 20 months). **(A)** Cell body area in the outer plexiform layer (OPL). **(B)** Cell body area in the inner plexiform layer (IPL). **(C)** Arborization area in the OPL. **(D)** Arborization area in the IPL. Data are shown as median with interquartile range. Each data point represents the individual morphometric value of a single Iba-1^+^ microglial cell, obtained from systematically acquired retinal images in which all microglial cells present were analysed. Statistical significance is shown as *p* < 0.05 (*), *p* < 0.01 (**), *p* < 0.001 (***), and *p* < 0.0001(****).

Further analysis revealed a significant decrease in soma size between 9 and both 12 and 15 months, followed by a renewed increase at 17 and 20 months. Comparisons from 12 months to 15, 17, and 20 months also showed consistent increases, highlighting a progressive increase in later stages. Additionally, soma size continued to rise between 15 and 17, and 15 and 20 months, with a further increase from 17 to 20 months, indicating ongoing structural remodelling (Figures 3, 5A and Supplementary Table S6).

##### 3.1.2.2 Inner plexiform layer (IPL)

In the IPL, soma size also changed significantly over time. Increases were evident between 6 and 9 months, as well as between 6 and 17 and 20 months. However, a decrease was observed between 6 and 12 months, mirroring the transient pattern seen in the OPL. Comparisons between 9 and 12, and 9 and 15 months revealed significant reductions, followed by a consistent increase from 12 to 15, 17, and 20 months. Similarly, soma size continued to rise from 15 to 17 and 20 months, with a final increase between 17 and 20 months, reflecting a late-stage increase of microglial soma size (Figures 3, 5B and Supplementary Table S7).

#### 3.1.3 Arborization area

##### 3.1.3.1 Outer plexiform layer (OPL)

At 20 months, the Iba-1^+^ arborization area was significantly reduced compared to earlier time points, including 6, 12, 15, and 17 months. In contrast, when compared to 9 months, a significant increase was observed. Additionally, arborization area at 9, 12, and 17 months was significantly lower than at 6 months. Conversely, significant increases were detected when comparing 9 months to 12, 15, and 17 months, as well as between 12 and 15 months a transient arborization area increase phase. Finally, a marked reduction in arborization area was observed at 17 months compared to 15 months (Figures 3, 5C and Supplementary Table S9).

##### 3.1.3.2 Inner plexiform layer (IPL)

At 20 months, the Iba-1^+^ arborization area was significantly lower than at earlier time points, including 6, 12, 15, and 17 months. Moreover, arborization area at 9, 12, and 17 months was reduced relative to 6 months. In contrast, significant increases were observed when comparing 9 months to 12, 15, and 17 months, indicating a transient arborization area increase phase likely to OPL. Finally, a pronounced decrease in arborization area was detected at 17 months compared to 15 months (Figures 3, 5D and Supplementary Table S10).

#### 3.1.4 Skeletonization

##### 3.1.4.1 Outer plexiform layer (OPL)

**Figure 6:**
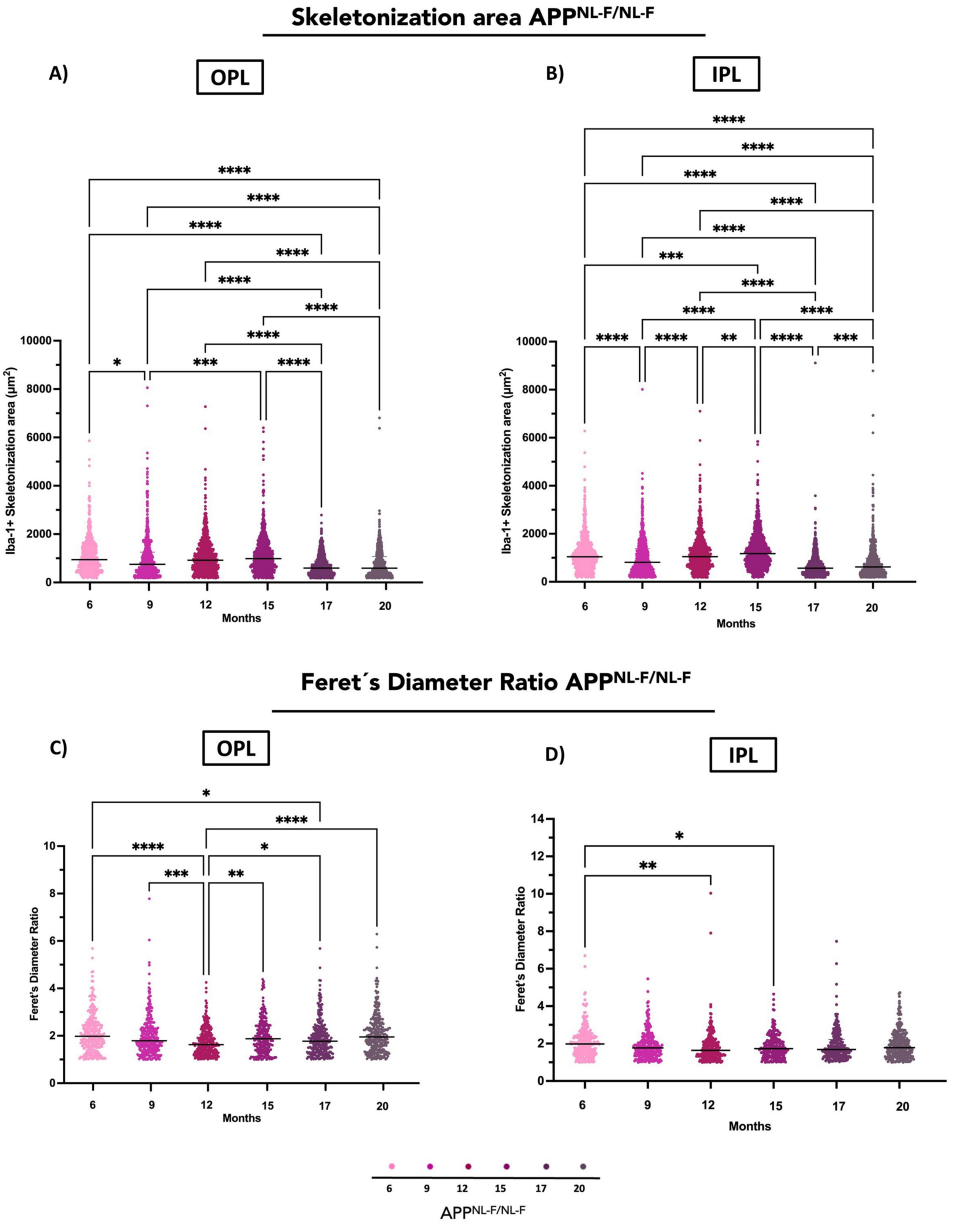
Quantitative analysis of Iba-1^+^ microglial morphology in the retina of *APP_NL-F/NL-F_* mice at different ages (6, 9, 12, 15, 17, and 20 months). **(A)** Skeletonization area in the outer plexiform layer (OPL). **(B)** Skeletonization area in the inner plexiform layer (IPL). **(C)** Feret’s diameter ratio in the OPL. **(D)** Feret’s diameter ratio in the IPL. Percentage changes (in red for increase, blue for decrease) are shown above significant comparisons. Data are shown as median with interquartile range. Each data point represents the individual morphometric value of a single Iba-1^+^ microglial cell, obtained from systematically acquired retinal images in which all microglial cells present were analyed. Statistical significance is shown as *p* < 0.05 (*), *p* < 0.01 (**), *p* < 0.001 (***), and *p* < 0.0001(****).

In APP^NL-F/NL-F^ mice, the Iba-1^+^ skeletonization area in the OPL exhibited significant temporal fluctuations. Compared to 6 months, the area was significantly reduced at 9 months and remained decreased at 17 and 20 months. In contrast, a significant increase was observed at 15 months compared to 6 months, suggesting a transient rise in skeletonization area. Further comparisons revealed that the skeletonization area at 17 and 20 months was significantly lower than at 9 months, while values at 12 and 15 months were significantly higher than at 9 months, indicating a biphasic pattern. When comparing 12 to 15 months, a significant increase was detected, whereas comparisons of 12 and 15 months to 17 and 20 months showed a significant decrease. Similarly, the area significantly decreased from 15 to 17 and 20 months, reflecting ongoing microglial remodelling in the aging APP^NL-F/NL-F^ retina (Figures 3, 6A and Supplementary Table S12).

##### 3.1.4.2 Inner plexiform layer (IPL)

In APP^NL-F/NL-F^ mice, the Iba-1^+^ skeletonization area in the IPL exhibited significant temporal variation. Compared to 6 months, this area was significantly reduced at 9 months, with a more pronounced decrease observed at 17 and 20 months, indicating an early and persistent decrease in microglial skeletonization area. Further comparisons showed that the skeletonization area at 17 and 20 months was also significantly lower than at 9 months. In contrast, values at 15 months were significantly higher than at 9 months suggesting a transient increase in microglial skeletonization area during midlife. Additionally, the skeletonization area at 12 was significantly higher than at 17 and 20 months, while a marked decline occurred when comparing 15 to 17 and 15 to 20 months (Figures 3, 6B and Supplementary Table S13).

#### 3.1.5 Feret’s diameter ratio (FDR)

##### 3.1.5.1 Outer plexiform layer (OPL)

A significant decrease in the FDR was observed when comparing early (6 months) and later time points, specifically at 12 months and 17 months. A similar reduction was also detected between 9 and 12 months, indicating a progressive thinning in the FDR in the OPL during this period. Interestingly, this trend reversed after 12 months, with a significant increase in FDR observed between 12 and 15 months, 12 and 17 months, and 12 and 20 months. These findings suggest a biphasic morphological change in the OPL (Figure 6C and Supplementary Table S15).

##### 3.1.5.2 Inner plexiform layer (IPL)

Notably, a significant decrease in FDR was observed between 6 and 12 months and 15 months, suggesting early structural alterations in the IPL (Figure 6D and Supplementary Table S16).

#### 3.1.6 Fluorescence intensity of Iba-1 staining in IPL and OPL

##### 3.1.6.1 Outer plexiform layer (OPL)

**Figure 7:**
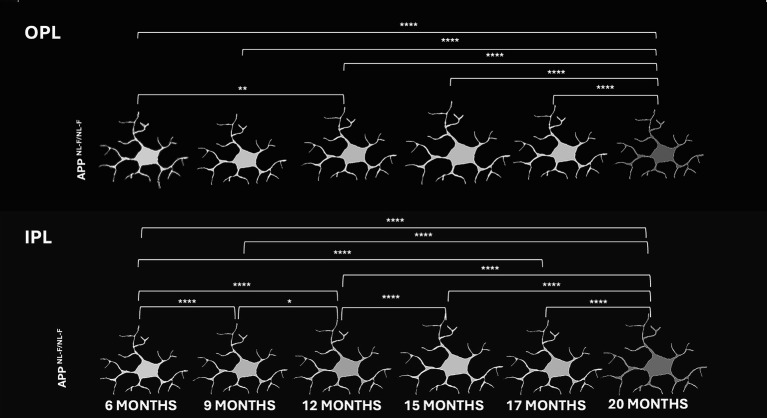
Representative schematic comparison of fluorescence intensity of Iba-1 + cells in the outer plexiform layer (OPL) and inner plexiform layer IPL of APP^NL-F/NL-F^ mice at 6, 9, 12, 15, 17, and 20 months of age. Whiter tones represent higher fluorescence intensity of Iba-1 staining, while darker gray tones indicate lower intensity, based on a grayscale range from 0 to 250. Statistical significance is shown as *p* < 0.05 (*), *p* < 0.01 (**), *p* < 0.001 (***), and *p* < 0.0001 (****).

A significant loss of Iba-1 fluorescence intensity was observed when comparing all earlier time points—6, 9, 12, 15, and 17 months—to 20 months, indicating a marked decline in microglial activation at the latest stage. Additionally, a significant reduction was also detected between 6 and 12 months, suggesting that the decrease of Iba-1 fluorescence intensity begins early and progresses over time (Figure 7 and Supplementary Table S18).

##### 3.1.6.2 Inner plexiform layer (IPL)

A significant decrease in Iba-1 fluorescence intensity was observed between 6 and 9, 12, 17, and 20 months, as well as between 9 and 17 months, 15 and 20 months, and 17 and 20 months. The comparison between 9 and 12 months also showed a decrease. In contrast, a marked increase in Iba-1 intensity was detected between 12 and 15 months and 12 and 17 months (Figure 7 and Supplementary Table S18).

### 3.2 Comparative temporal study between the WT and the APP^NL-F/NL-F^ at different time points

#### 3.2.1 Number of Iba-1+ cells

##### 3.2.1.1 Outer segment layer (OS)

**Figure 8:**
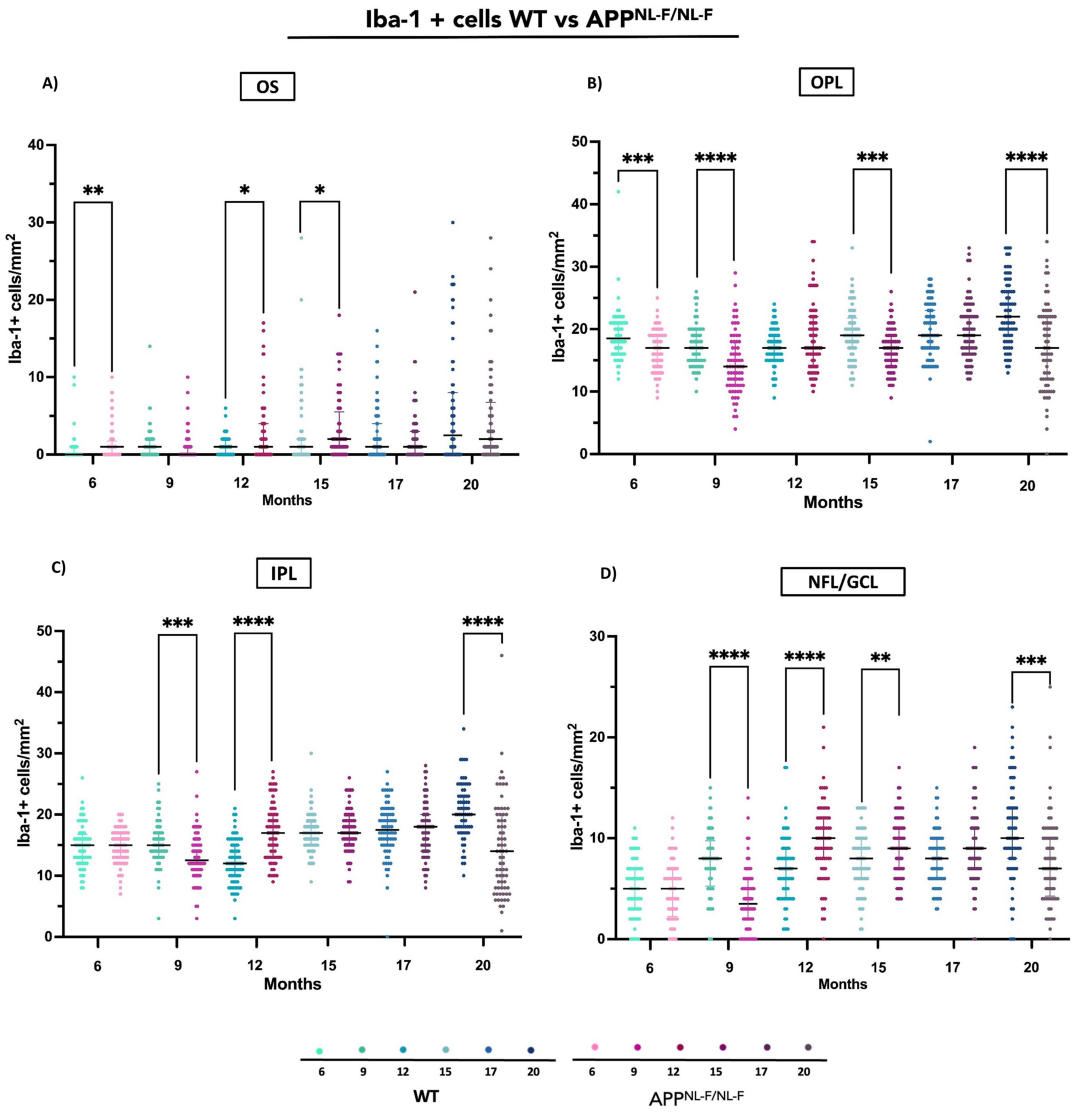
Quantification of Iba-1^+^ cells in different retinal layers (OS, OPL, IPL, NFL/GCL) across age groups in wild-type (WT) and APP^NLF-NLF^ groups. **(A)** Outer segment (OS), **(B)** Outer plexiform layer (OPL), **(C)** Inner plexiform layer (IPL), and **(D)** Nerve fiber layer/ganglion cell layer (NFL–GCL). Data are shown as median with interquartile range. Each data point represents the total number of Iba-1^+^ microglial cells identified within a systematically defined anatomical field of 0.1502 mm^2^, in which all microglial cells present were counted. Statistical significance is shown as *p* < 0.05 (*), *p* < 0.01 (**), *p* < 0.001 (***), and *p* < 0.0001(****).

APP^NL-F/NL-F^ mice exhibited significantly higher Iba-1^+^ microglial cell densities compared to WT at several time points. Notable differences were detected at 6 months, that remained evident at 12 and 15 months indicating early and sustained microglial activation in this layer (Figure 8A and Supplementary Table S5).

##### 3.2.1.2 Outer plexiform layer (OPL)

In contrast, APP^NL-F/NL-F^ retinas showed a marked reduction in microglial density relative to WT at 6, 9, 15 and 20 months (Figures 3, 8B and Supplementary Table S5).

##### 3.2.1.3 Inner plexiform layer (IPL)

Significant differences were also observed in the IPL. APP^NL-F/NL-F^ mice had lower Iba-1^+^ cell counts at 9 and 20 months compared to WT. Interestingly, at 12 months, the APP^NL-F/NL-F^ group showed a pronounced increase in microglial density, suggesting a transient peak in activation (Figures 3, 8C and Supplementary Table S5).

##### 3.2.1.4 Nerve fiber layer/ ganglion cell layer (NFL/GC)

In the NFL/GCL, APP^NL-F/NL-F^ retinas displayed significantly reduced microglial density at 9 and 20 months relative to WT. Conversely, elevated significantly levels were observed at 12 and 15 months (Figure 8D and Supplementary Table S5).

#### 3.2.2 Soma size

##### 3.2.2.1 Outer plexiform layer (OPL)

**Figure 9:**
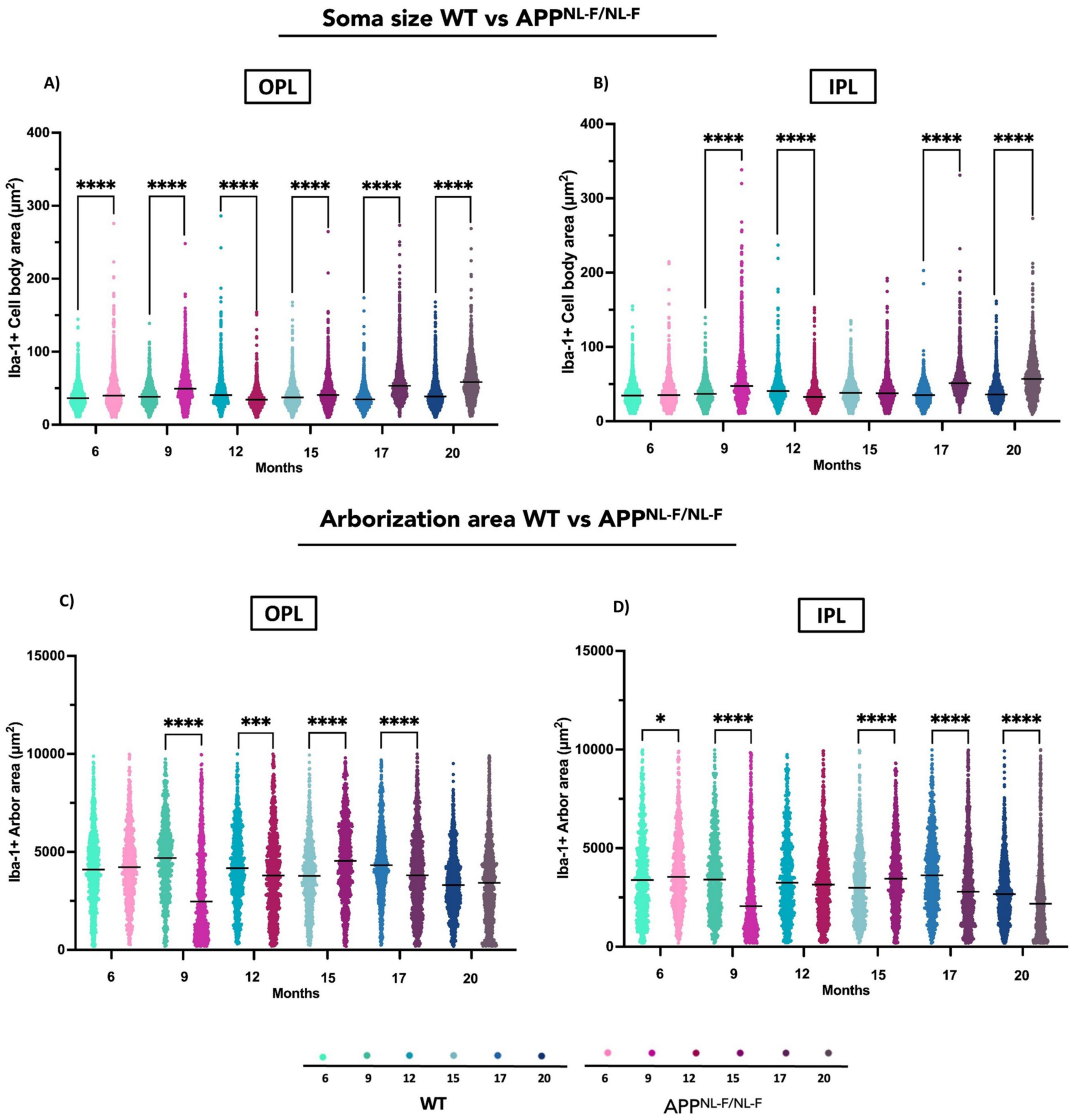
Comparative analysis of Iba-1^+^ microglial morphology between wild-type (WT) and APP^NL-F/NL-F^ mice at different ages (6, 9, 12, 15, 17, and 20 months). **(A)** Cell body area in the outer plexiform layer (OPL). **(B)** Cell body area in the inner plexiform layer (IPL). **(C)** Arborization area in the OPL. **(D)** Arborization area in the IPL. Data are shown as median with interquartile range. Each data point represents the individual morphometric value of a single Iba-1^+^ microglial cell, obtained from systematically acquired retinal images in which all microglial cells present were analysed. Statistical significance is shown as *p* < 0.05 (*), *p* < 0.01 (**), *p* < 0.001 (***), and *p* < 0.0001(****).

Significant differences in Iba1^+^ soma size were observed between WT and APP^NL-F/NL-F^ mice across all examined time points. The transgenic group exhibited markedly larger soma sizes at 6, 9, 15, 17, and 20 months, indicating sustained microglial hypertrophy. Interestingly, at 12 months, APP^NL-F/NL-F^ mice showed a significant reduction in soma size compared to WT, suggesting a transient morphological shift (Figures 3, 9A and Supplementary Table S8).

##### 3.2.2.2 Inner plexiform layer (IPL)

In the IPL, APP^NL-F/NL-F^ mice also displayed significantly enlarged soma sizes at 9, 17, and 20 months relative to WT. However, a notable decrease was observed at 12 months, mirroring the pattern seen in the OPL. These findings highlight a complex, time-dependent remodelling of microglial morphology in the APP^NL-F/NL-F^ model, characterized by early and late-stage hypertrophy interspersed with transient decrease, consistent with chronic and evolving microglial activation in Alzheimer’s disease pathology (Figures 3, 9B and Supplementary Table S8).

#### 3.2.3 Arborization area

##### 3.2.3.1 Outer plexiform layer (OPL)

At 9, 12 and 17 months, the arborization area was significantly reduced in the APP^NL-F/NL-F^ group compared to WT, indicating early and sustained microglial activation. In contrast, at 15 months, the APP model exhibited a significantly larger arborization area suggesting a transient phase with less activation (Figures 3, 9C and Supplementary Table S11).

##### 3.2.3.2 Inner plexiform layer (IPL)

In the IPL, APP^NL-F/NL-F^ mice showed notable alterations in Iba-1^+^ arborization area compared to WT controls at several time points. At 6 months, and more prominently at 9, 17, and 20 months, the transgenic group displayed a significant reduction in arborization area similar to OPL. Conversely, at 15 months, APP^NL-F/NL-F^ mice exhibited a pronounced increase in arborization area relative to WT like to OPL (Figures 3, 9D and Supplementary Table S11).

#### 3.2.4 Skeletonization

##### 3.2.4.1 Outer plexiform layer (OPL)

**Figure 10:**
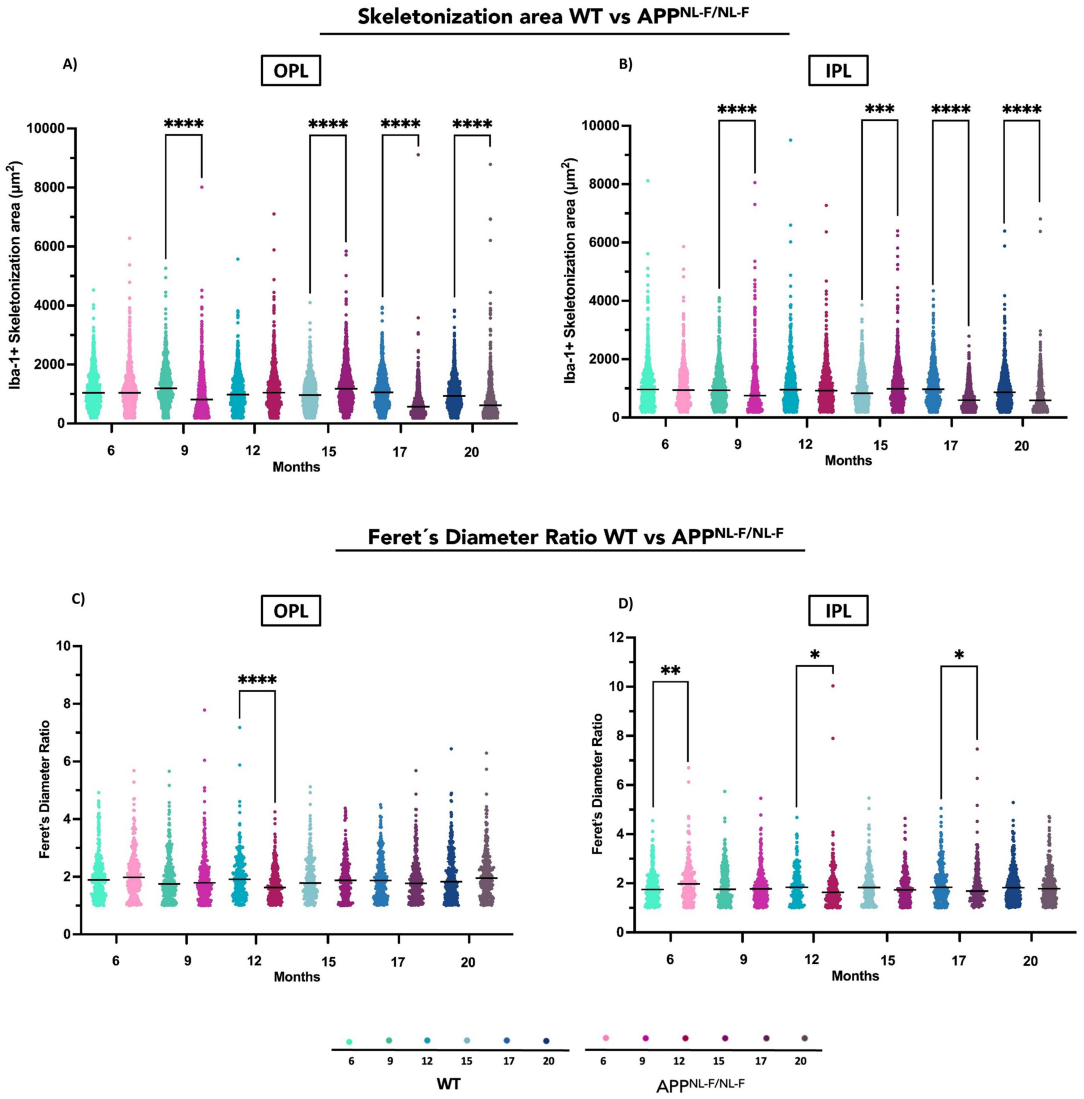
Comparative analysis of Iba-1^+^ microglial morphology between wild-type (WT) and APP^NL-F/NL-F^ mice at different ages (6, 9, 12, 15, 17, and 20 months). **(A)** Skeletonization area in the outer plexiform layer (OPL). **(B)** Skeletonization area in the inner plexiform layer (IPL). **(C)** Feret’s diameter ratio in the OPL. **(D)** Feret’s diameter ratio in the IPL. Data are shown as median with interquartile range. Each data point represents the individual morphometric value of a single Iba-1^+^ microglial cell, obtained from systematically acquired retinal images in which all microglial cells present were analyzed. Statistical significance is shown as *p* < 0.05 (*), *p* < 0.01 (**), *p* < 0.001 (***), and *p* < 0.0001 (****).

APP^NL-F/NL-F^ mice demonstrated significant deviations in Iba-1^+^ skeletonization area relative to WT at several time points. A marked decrease in this area was evident at 9, 17, and 20 months, reflecting a sustained simplification of microglial complexity. In contrast, at 15 months, the transgenic group exhibited a notable increase in skeletonization area, suggesting a temporary phase of heightened microglial complexity that diverges from the overall trend of reduction (Figures 3, 10A and Supplementary Table S14).

##### 3.2.4.2 Inner plexiform layer (IPL)

A similar pattern was observed in the IPL, with significant reductions in skeletonization area at 9, 17, and 20 months in APP^NL-F/NL-F^ mice compared to WT. However, at 15 months, the increase in skeletonization area was also present but slightly less pronounced, indicating a comparable yet more moderate transient response in microglial structure in comparison with OPL (Figures 3, 10B and Supplementary Table S14).

#### 3.2.5 Feret’s diameter ratio (FDR)

##### 3.2.5.1 Outer plexiform layer (OPL)

Both groups exhibited temporal changes in FDR; however, a statistically significant difference was observed at 12 months, where APP^NL-F/NL-F^ mice showed a markedly lower FDR compared to WT controls (Figure 10C and Supplementary Table S17).

##### 3.2.5.2 Inner plexiform layer (IPL)

At 6 months, APP^NL-F/NL-F^ mice exhibited a significantly higher FDR compared to WT controls, suggesting early structural alterations in the IPL. Additional significant decrease in FDR in the transgenic mouse related to WT were observed at 12 months and 17 months (Figure 10D and Supplementary Table S17).

#### 3.2.6 Fluorescence intensity of Iba-1 staining in IPL and OPL

##### 3.2.6.1 Outer plexiform layer (OPL)

**Figure 11:**
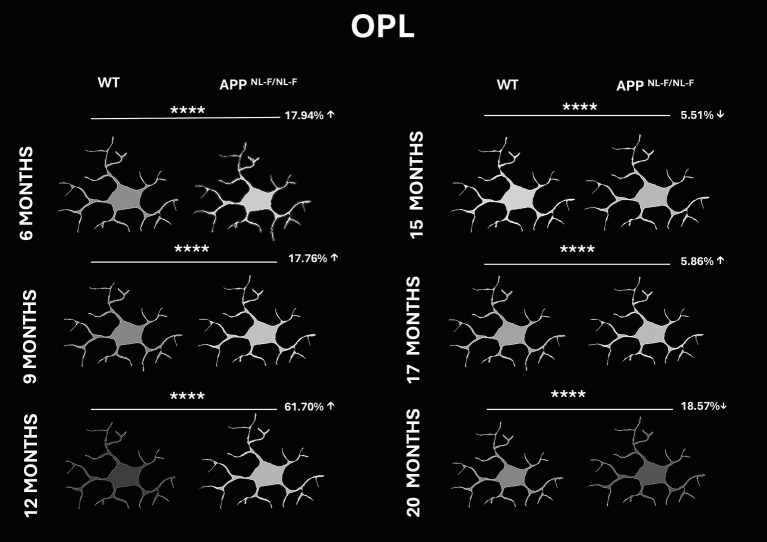
Representative schematic comparison of fluorescence intensity of Iba-1 + cells in the outer plexiform layer (OPL) of wild-type (WT) and APP^NL-F/NL-F^ mice at 6, 9, 12, 15, 17, and 20 months of age. Percentage values indicate the relative increase (↑) or decrease (↓) in the analyzsed parameter compared to WT controls. Whiter tones represent higher fluorescence intensity, while darker gray tones indicate lower intensity, based on a grayscale range from 0 to 250. Statistical significance is shown as *p* < 0.0001 (****).

Significant differences in Iba-1 signal intensity were observed between WT and APP^NL-F/NL-F^ mice across all analysed time points. During the early stages, APP mice exhibited higher fluorescence intensity compared to WT, with increases of 17.94 and 17.76% at 6 and 9 months, respectively. A pronounced elevation was noted at 12 months, where APP^NL-F/NL-F^ mice showed a 61.7% increase relative to WT. However, this trend reversed at later stages. At 15 months, APP mice displayed a 5.51% decrease in signal intensity compared to WT. At 17 months, the intensity was slightly elevated by 5.86%, but by 20 months, APP^NL-F/NL-F^ mice exhibited a notable 18.57% decrease relative to WT, suggesting a late-stage decrease in OPL microglial activation in the Alzheimer’s disease model (Figure 11 and Supplementary Table S18).

##### 3.2.6.2 Inner plexiform layer (IPL)

**Figure 12:**
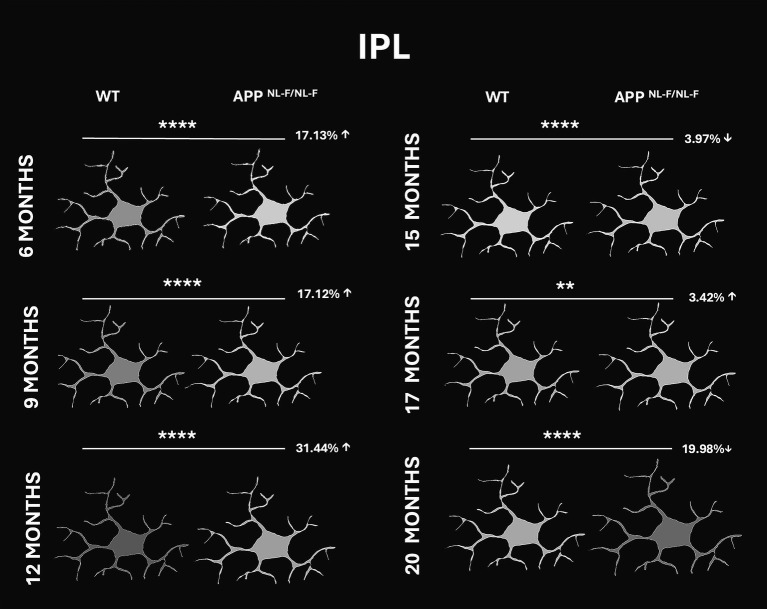
Representative schematic comparison of fluorescence intensity of Iba-1^+^ cells in the inner plexiform layer (IPL) of wild-type (WT) and APP^NL-F/NL-F^ mice at 6, 9, 12, 15, 17, and 20 months of age. Percentage values indicate the relative increase (↑) or decrease (↓) in the analyzed parameter compared to WT controls. Whiter tones represent higher fluorescence intensity, while darker gray tones indicate lower intensity, based on a grayscale range from 0 to 250. Statistical significance is shown as *p* < 0.01 (**) and *p* < 0.0001(***).

At 6 and 9 months, APP^NL-F/NL-F^ mice exhibited a 17.13 and 17.12% increase in Iba-1 fluorescence intensity, respectively, compared to WT controls. This elevation became more pronounced at 12 months, with a 31.44% increase, indicating progressive microglial activation associated with early stages of AD. By 15 months, a 3.97% decrease in signal intensity was observed in APP mice relative to WT, followed by a slight increase of 3.42% at 17 months. Notably, at 20 months, APP mice showed a marked reduction of 19.98% compared to WT, suggesting a late-stage decrease in microglial activity within the IPL (Figure 12 and Supplementary Table S18).

## 4 Discussion

In the pigmented C57BL/6 J mouse model, resting retinal microglia exhibit a regular mosaic distribution and a highly ramified morphology, with processes predominantly located in the IPL and OPL (Santos et al., 2008). Aging is associated with progressive microglial activation, peaking at 20 months. This process involves an increase in microglial cell number across all retinal layers, enlargement of soma size (maximal in the OPL at 12 months and in the IPL at 15 months), and a reduction in arborization and skeletonization. A transient phase between 15 and 17 months may represent an intermediate state between early activation and advanced dysfunction.

Iba1 + signal intensity decreases from 12 months onward, peaking at 15 months and subsequently declining until 20 months. The thickening of the OPL observed via OCT imaging may be associated with increased microglial cell density and soma size, probably linked to synaptic stripping (de Hoz et al., 2013; Kettenmann et al., 2013). Previous studies in albino mouse models have similarly reported microglial activation at 15 months, with increased cell numbers in the photoreceptor layer, enlarged soma areas across multiple layers (OPL, IPL, NFL/ GCL) and vertical processes spanning from the OS to the OPL (Ramírez et al., 2020). In the CX3CR1+/GFP model, a mild but significant increase in microglial cells in the IPL and OPL has been observed, accompanied by reduced arborization and decreased process motility in aged microglia (Damani et al., 2011).

These morphological changes reflect a functional transition from a neuroprotective M2 phenotype to a pro-inflammatory M1 state, observed in both control and APP^NL-F/NL^ models, with the latter exhibiting an accelerated progression due to chronic exposure to Aβ and p-tau. This microglial polarization pattern may contribute to progressive neuronal degeneration.

Transgenic models such as Tg2576 and 5xFAD show activated microglia in the IPL and OPL (Tisi et al., 2025), with amoeboid morphology and a neurodegenerative profile (Zhang et al., 2021; Okhalnikov et al., 2023) In the 3xTg-AD model, early expression of anti-inflammatory genes (e.g., Ym1, CD206) is followed by a decline as the disease progresses, while pro-inflammatory genes (e.g., iNOS, IL-1β) increase (Grimaldi et al., 2018). Aged or chronically stimulated microglia exhibit impaired phagocytic capacity, reduced responsiveness to inflammatory signals, and adopt a senescent gene expression profile states (Keren-Shaul et al., 2017; Streit et al., 2009) and impaired responsiveness to anti-inflammatory cytokines (Daria et al., 2017). In the hippocampus of aged mice, a senescence-associated secretory phenotype has been described, characterized by hyperphagocytosis of excitatory synapses, contributing to cognitive decline (Liu et al., 2025).

In human retinas affected by AD, reduced colocalization of microglia with Aβ deposits has been reported, indicating microglial dysfunction and impaired phagocytic capacity (Swanson et al., 2020). This dysfunction includes inappropriate activation and loss of neuroprotective function (Streit et al., 2009; Mosher and Wyss-Coray, 2014; Xu et al., 2022; Streit, 2004; Streit et al., 2020), and has also been described in the brain as a progressive process involving energy depletion, loss of ramification, and release of neurotoxic mediators (Sarlus and Heneka, 2017).

In the APP^NL-F/NL-F^ mouse model, elevated Aβ42 levels are detected in the cortex and hippocampus by 6 months, forming plaques surrounded by microglia and astrocytes (Sasaguri et al., 2017). In the retina, we observed a biphasic pattern of Iba1^+^ microglial cell density: an initial increase from 6 to 17 months, followed by a decline at 20 months, suggesting early activation and later functional exhaustion. Similar early activation patterns have been reported in 5xFAD (Zhang et al., 2021; Lim et al., 2020), 3xTg-AD (Grimaldi et al., 2018; Salobrar-García et al., 2020), and APP/PS1(Ning et al., 2008) models, showing early cortical neuroinflammation and synaptic loss, accompanied by transcriptional profiles of activated microglia, including the Disease-Associated Microglia (DAM) phenotype marked by ApoE, Trem2, and Cst7 expression (Keren-Shaul et al., 2017; Deczkowska et al., 2018).

Microglial uptake of phosphorylated tau (P-tau) intensifies with disease progression, with early detection of tau-binding ligand bTVBT2 in 50% of Iba1^+^ cells at 3 months, increasing between 9 and 12 months (Nuñez-Diaz et al., 2024). Interestingly, P-tau uptake levels between 12 and 18 months were comparable to WT mice, suggesting a physiological role in early stages and a pathological role later.

Soma size also followed a biphasic pattern, increasing between 6–9 and 15–20 months, then declining at 20 months. This may reflect distinct activation states. Notably, microglial activation in this model begins earlier (6 months) than in APP/PS1 mice, where activation is reported at 8 or even 27 months (Ning et al., 2008). Early activation may be driven by the need to phagocytose P-tau, which accumulates from 3 months of age (Nuñez-Diaz et al., 2024). In the App ^NL-G-F^ model, activation is observed at 3 months, with enlarged somas and retracted processes (Vandenabeele et al., 2021), likely driven by soluble Aβ accumulation, as also shown the brain (Hong et al., 2016).

Arborization and skeletonization showed layer-specific changes. In the OPL, a biphasic decrease was interrupted by a transient increase between 12 and 15 months; in the IPL, a gradual decline was observed, also with a transient increase in the same window. These changes suggest progressive loss of morphological complexity. OCT analysis revealed thinning of both plexiform layers, potentially linked to reduced soma size, arborization, and microglial redistribution across retinal layers (Sánchez-Puebla et al., 2024a), probably in response to oligomeric Aβ accumulation.

The IPL exhibited earlier and more sustained activation than the OPL, consistent with early ganglion cell degeneration in 3xTg-AD (Grimaldi et al., 2018) and APP/PS1 AD models (Georgevsky et al., 2019),and increased microglial density in the IPL and RNFL in 5xFAD mice as early as 4 months of age months (Zhang et al., 2021). The IPL is also one of the most affected layers in terms of thickness loss in the APP^NL-F/NL-F^ model (Sánchez-Puebla et al., 2024a), paralleling Aβ and p-tau accumulation in human AD retinas (La Morgia et al., 2017).

In our study, FDR decreased in both IPL and OPL until 12 months, followed by a peak increase from 15 months onward, indicating a shift from rounded to less amoeboid morphology. Iba1^+^ signal intensity also declined progressively, with a transient peak between 12 and 17 months, possibly reflecting overexpression during activation followed by reduced expression in advanced stages. A similar pattern of increased F4/80 immunoreactivity has been reported in APP/PS1 mice between 12 and 16 months compared to a control group (Perez et al., 2009). Recent findings suggest that Iba1 may not be universally expressed across all microglial subpopulations, with some subsets expressing CD68 or P2RY12 but lacking Iba1 (Lier et al., 2019; Waller et al., 2019). Thus, changes in Iba1 signal may reflect alterations in microglial behavior, particularly cytoskeletal activity or increased motility, rather than cell number. This aligns with Iba1’s role as an actin-binding protein involved in cytoskeletal remodelling (Franco-Bocanegra et al., 2019; Sasaki et al., 2001), and with reports of elevated P2RY12 expression during specific activation states (Nuñez-Diaz et al., 2024). Such mechanisms could be account for the age-related and pathology-associated variations in Iba1 signal intensity observed in both wild-type and APP^NL-F/NL-F^ mice in our analysis.

Comparative analysis between WT and APP^NL-F/NL-F^ mice reveals distinct microglial dynamics across retinal layers and time points. In the APP^NL-F/NL-F^ model, microglial cell numbers significantly increase in the OS layer at 6, 12, and 15 months, while other layers show early reductions with transient increases in the IPL (12 months) and RNFL-GCL (12–15 months). The RNFL thickening observed at 17 months by OCT may reflect this cellular increase (Sánchez-Puebla et al., 2024a).

Microglial soma size is consistently larger in APP^NL-F/NL-F^ mice than WT, indicating sustained activation typical of chronic inflammation (Vandenabeele et al., 2021). A temporary reduction at 12 months may represent a transitional phase. Similar early activation has been reported in 5xFAD AD mice, correlating with increased retinal thickness (Zhang et al., 2021).

APP^NL-F/NL-F^ mice also show reduced arborization in the OPL and IPL, with earlier and more pronounced changes in the IPL, suggesting stronger activation. Skeletonization is significantly reduced at 9, 17, and 20 months, indicating less ramified microglia. These morphological changes, along with soma size reduction at 12 months, may contribute to inner retinal thinning in OCT observed over time in APP ^NL-F/NL-F^ mice compared to WT (Sánchez-Puebla et al., 2024b). This fact is consistent with neurodegeneration reported in 3xTg-AD (Georgevsky et al., 2019) and APP/PS1 models (Xu et al., 2022).

FDR is lower in APP^NL-F/NL-F^ mice from early stages, increasing after 15 months, reflecting a shift toward an amoeboid morphology with reduced arborization. Iba1^+^ signal intensity declines progressively from 6 to 20 months, with a transient peak between 12–17 months, possibly indicating temporary overexpression followed by dysfunction.

Overall, APP^NL-F/NL-F^ mice exhibit a dynamic progression of retinal microglial activation, remodelling, and late-stage dysfunction, paralleling cerebral pathology in this model (Saito et al., 2014) and other AD models.

The retina mirrors cerebral neuroinflammation in multiple AD models, supporting its role as an early, non-invasive biomarker (Salobrar-García et al., 2021; Grimaldi et al., 2018). Microglial activation, p-tau uptake (Nuñez-Diaz et al., 2024), and reduced morphological complexity in APP^NL-F/NL-F^ mice highlight this potential. Human studies using OCT, autofluorescence, and fluorescent ligands have begun exploring this approach (Koronyo et al., 2017; Hadoux et al., 2019). Preclinical validation facilitates the development of accessible diagnostic tools, particularly for presymptomatic detection.

One of the limitations of this study is that it could not be conducted as a purely longitudinal analysis, since tissue processing required sacrificing animals at each time point. Nevertheless, this is the first study to characterize microglial morphological changes in the APP^NL-F/NL-F^ model across an extended temporal window. Moreover, the comprehensive analysis performed here—spanning multiple retinal layers, two experimental groups, and six time points—was made possible by an automated system specifically developed for microglial quantification and morphological characterization. This approach substantially reduced analysis time and improved measurement accuracy and standardization compared to manual methods (Sánchez-Puebla et al., 2025).

In conclusion, retinal microglia show age-related morphometric changes in C57BL/6 mice, progressive activation with increased cell numbers, soma enlargement, and reduced arborization, while APP^NL-F/NL-F^ mice exhibit earlier, more intense activation from 6 months, with biphasic phases and later dysfunction, paralleling cerebral pathology. Using an automated morphometric pipeline ensured consistent evaluation across all samples and minimized operator-dependent variability. Overall, retinal analysis emerges as a promising peripheral biomarker for early AD detection, given its accessibility and correlation with OCT structural changes.

## Data Availability

The original contributions presented in the study are included in the article/Supplementary material, further inquiries can be directed to the corresponding author. The software used in this article is registered with the Intellectual Property Registry as the computer program MorphoSomas (registration number M-002548/2025, entry 16/2025/5344). Further inquiries regarding the software can be directed to the corresponding author.
