# Supplementary material for: Early and progressive retinal microglial changes in APPNL-F/NL-F mouse model of Alzheimer’s disease revealed by an automated image analysis software
Source: Front Aging Neurosci. 2026 Jan 15;17:1712480. doi: 10.3389/fnagi.2025.1712480 (PMC12852431; doi:10.3389/fnagi.2025.1712480)
Supplement: Supplementary file 1 [file Data_Sheet_1.docx]

Supplementary Material

## Temporal Study of the WT Mice

### Number of Iba-1+ cells

**Outer segment layer (OS)**

Quantification of Iba-1⁺ microglial cells in the OS of WT retinas revealed a time-dependent increase in cell density. Significant increases were observed between 20 months and all earlier time points (6, 9, 12, 15, and 17 months. Additionally, a moderate but significant increase was detected between 6 and 15 months (Figure S1A and Table S1).

**Outer plexiform layer (OPL)**

In the OPL, microglial density also increased with age in WT retinas. The 20-month group showed significantly higher values compared to 6, 9, 12, 15 months and 17 months. Comparisons between intermediate time points revealed additional increases: 9 vs.17 months and 12vs. 15 months, as well as 12 vs.17 months, suggesting a gradual buildup of microglial presence (Figure 3, Figure S1B and Table S2).

**Inner plexiform layer (IPL)**

A significant rise in Iba-1⁺ microglial cell density was observed at 20 months when compared to all earlier time points (6, 9, 12, 15, and 17 months). Additionally, significant increases were detected at 15 and 17 months relative to 6 months, however when we compare the last with 12 months values were significantly lower. When we compared between 9 with 12 months we found a significant decrease in the number of Iba-1⁺ cells, and a significant increase at 17 months. Moreover, a significant increase was observed between 12 and both 15 and 17 months (Figure 3, Figure S1C and Table S3).

**Nerve fiber layer/ Ganglion cell layer (NFL/GC).**

A statistically significant increase in number Iba-1⁺ cells values was observed over time. At 20 months, the values were markedly elevated compared to all preceding time points (6, 9, 12, 15, and 17 months). Additionally, significant increases were detected at 9, 12 , 15 and 17 months compared to 6 months (Figure S1D and Table S4).


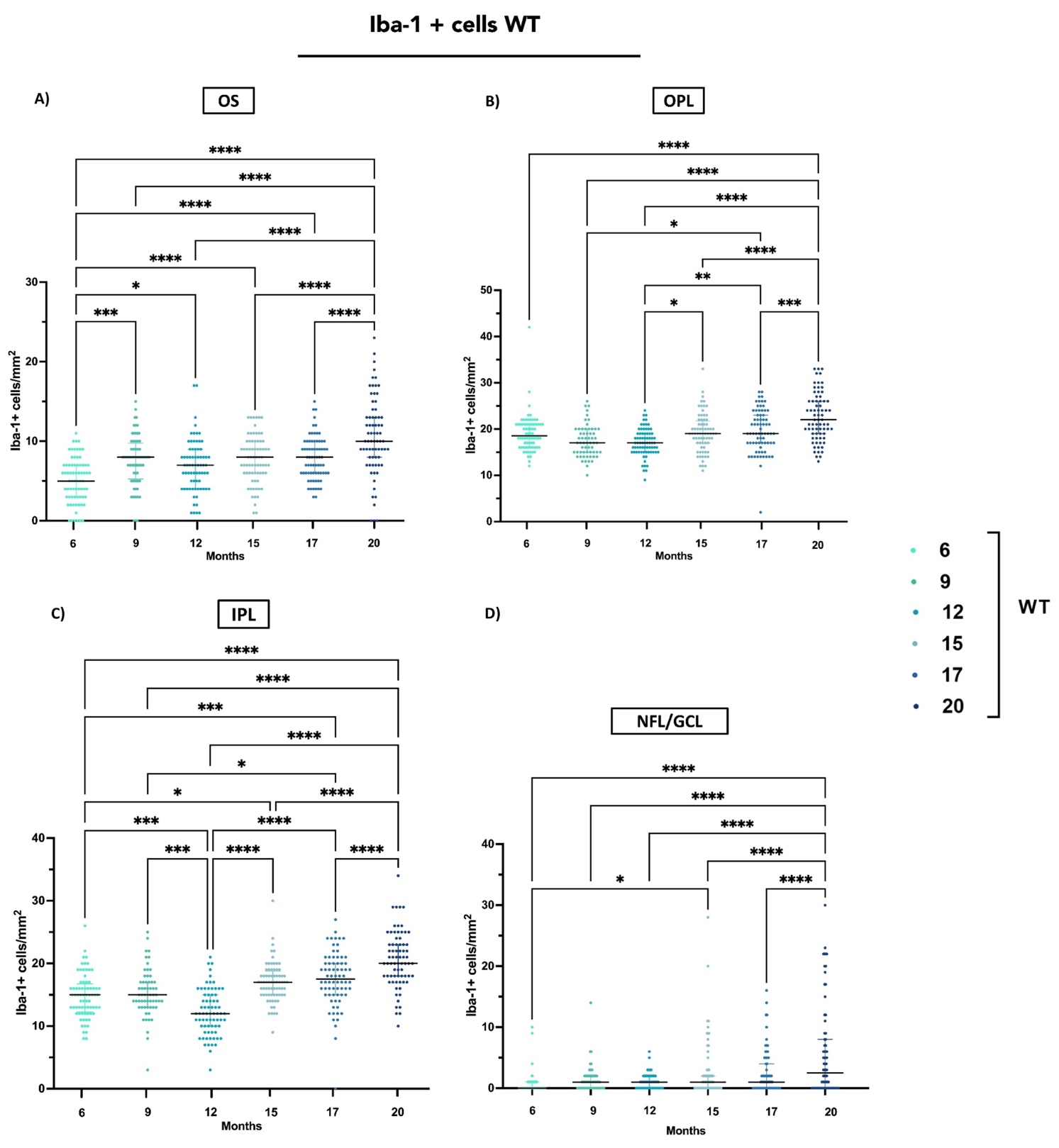


Figure S1. Quantification of Iba-1 + cells in different retinal layers (OS, OPL, IPL, NFL/GCL) across age groups in wild-type (WT) group. A) outer segment (OS), (B) Outer plexiform layer (OPL), (C) Inner plexiform layer (IPL), and (D) Nerve fiber layer/ganglion cell layer (NFL–GCL). *Data are shown as median with interquartile range. Each data point represents the total number of Iba-1⁺ microglial cells identified within a systematically defined anatomical field of 0.1502 mm², in which all microglial cells present were counted.* Statistical significance is shown as p < 0.05 (*), p < 0.01 (**), p < 0.001 (***), and p < 0.0001(****).

### Soma size

**Outer plexiform layer (OPL)**

In the OPL, soma size of Iba-1⁺ microglial cells exhibited dynamic changes over time. A pronounced significative enlargement was observed between 6 and 20 months, as well as between 6 and 12 months, suggesting early morphological increase. Additional soma size increases were detected between 9 and 12 months and between 9 and 20 months. In contrast, a reduction in soma size was noted between 9 and 17 months. Further decreases were evident when comparing 12 months with 15, 17 and 20 months indicating a change in microglial morphology during mid to late aging. Late-stage changes were also observed between 15 vs. 17 months, where we found a decrease in soma size. However, a statistically significant increase was found at 15 vs. 20 months, and 17 vs. 20 months (Figure 3, Figure S2A and Table S6).

Inner plexiform layer (IPL)

In the IPL, soma size also varied significantly with age. Marked increases were observed between 6 and 12 months, 6 and 15 months, and 6 and 20 months, reflecting early and sustained microglial activation. A similar pattern was seen between 9 and 12 months, while a significant reduction was found between 9 and 17 months. Subsequent comparisons revealed further decreases between 12 and 15, 17, and 20 months, suggesting a decline in soma size following the initial peak. Notably, a reduction was also observed between 15 and 17 months, followed by a significant increase from 17 to 20 months, indicating late-stage morphological change (Figure 3, Figure S2B and Table S7).

### Arborization area

**Outer plexiform layer (OPL)**

In the OPL, WT mice exhibited significantly increased Iba-1⁺ arborization areas at 9 and 17 months compared to 6 months. Interestingly, a reduction was observed at 15 months relative to 6 months. When comparing 9 months to later time points, a significant decrease in arborization area was found at 12, 15, and 20 months. Similarly, arborization area was reduced at 15 and 20 months when compared to 12 months. Notably, an increase in arborization area was detected between 15 and 17 months. However, by 20 months, arborization area was significantly reduced compared to all earlier time points (6, 9, 12, 15, and 17 months), indicating a marked decline in microglial arborization area at advanced age (Figure 3, Figure S2C and Table S9).

Inner plexiform layer (IPL)

In the IPL of WT retinas, the Iba-1⁺arborization area showed significant age-related alterations. At 20 months, arborization area was markedly reduced compared to all earlier time points (6, 9, 12, 15, and 17 months), indicating a substantial decline in microglial arborization area at advanced age. Additionally, at 15 months, a significant reduction was observed relative to 6, 9, and 12 months. The only increase detected occurred between 15 and 17 months, suggest the presence of a transient phase of increased arborization extension prior to the eventual decline in process complexity (Figure 3, Figure S2D and Table S10).

### Skeletonization

**Outer plexiform layer (OPL)**

In WT mice, the Iba-1⁺ skeletonization area in the OPL exhibited significant temporal modulation. Compared to 6 months, the skeletonization area was markedly increased at 9 months and remained elevated at 20 months. However, when compared 6 to 15 months, the area showed a significant decrease, indicating a peak followed by a decrease. Further comparisons revealed that skeletonization area at 12, 15, 17, and 20 months was significantly lower than at 9 months, suggesting that the increase observed at 9 months was not sustained. Interestingly, a transient increase was observed between 15 and 17 months, followed by a significant reduction from 17 to 20 months, highlighting a dynamic remodelling of microglial structure during aging (Figure 3, Figure S2E and Table S12).

Inner plexiform layer (IPL)

A similar pattern was observed in the IPL. Compared to 6 months, the skeletonization area significantly decreased at 15 months and remained decrease at 20 months. Additionally, a significant reduction was evident when comparing 12 to 20 months. Additionally, skeletonization area at 12 months was significantly higher than at both 15 and 20 months. A modest significant increase was detected between 15 and 17 months, followed by a significant decrease from 17 to 20 months, again suggesting dynamic age-related changes in microglial structure (Figure 3, Figure S2F and Table S13).


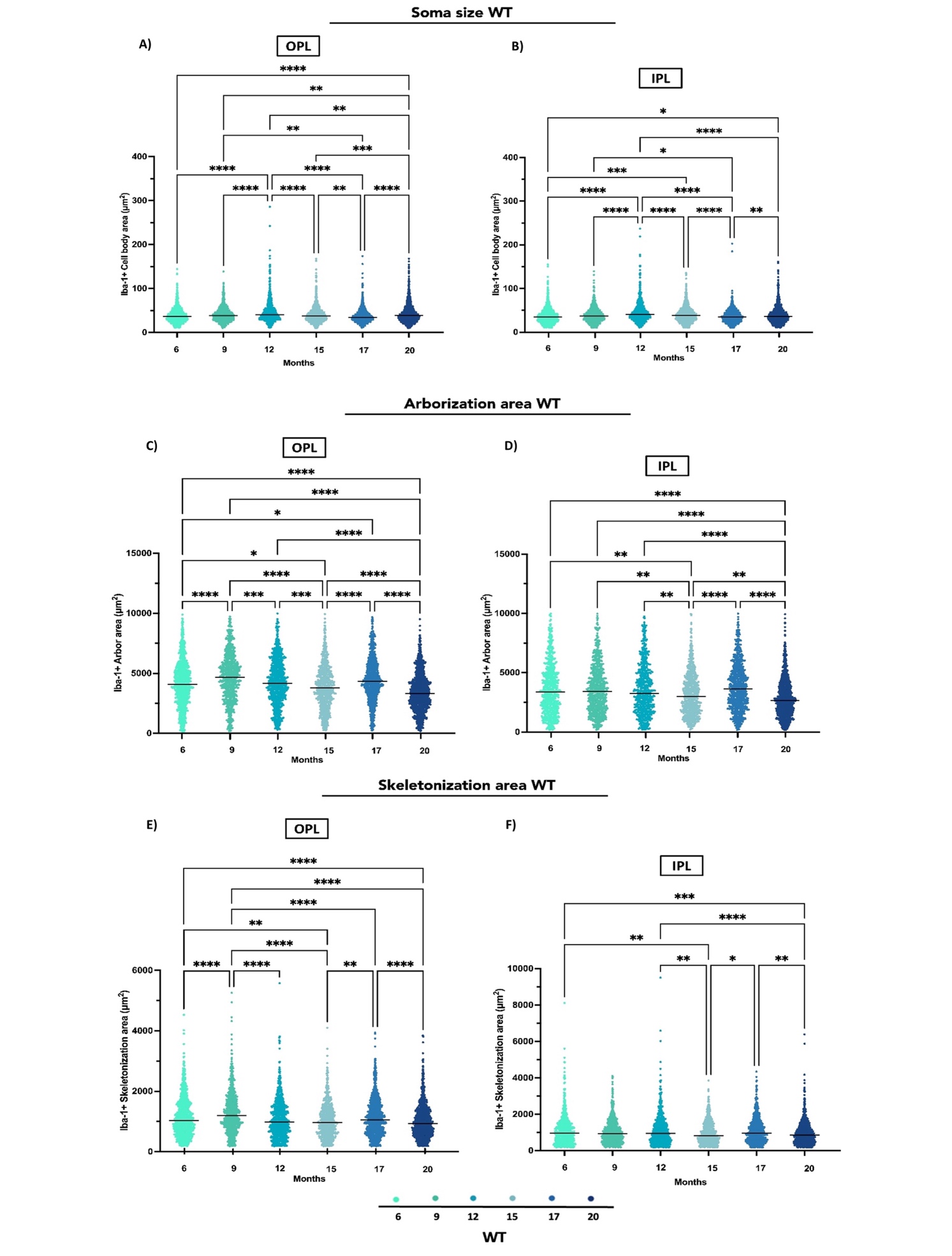


**Figures S2. Quantitative analysis of Iba-1⁺ microglial morphology in the retina of wild-type (WT) mice at different ages (6, 9, 12, 15, 17, and 20 months). (A) Cell body area in the outer plexiform layer (OPL). (B) Cell body area in the inner plexiform layer (IPL). (C) Arborization area in the OPL. (D) Arborization area in the IPL. (E) Skeletonization area in the OPL. (F) Skeletonization area in the IPL.** *Data are shown as median with interquartile range. Each data point represents the individual morphometric value of a single Iba-1⁺ microglial cell, obtained from systematically acquired retinal images in which all microglial cells present were analysed***. Statistical significance is shown as p < 0.05 (*), p < 0.01 (**), p < 0.001 (***), and p < 0.0001(****).**

### Feret´s Diameter Ratio (FDR)

Neither OPL nor IPL layers showed any statistical changes in FDR (Table S15 and Table S16)

### Fluorescence intensity of Iba-1 staining in IPL and OPL.

**Outer Plexiform Layer (OPL)**

Quantitative analysis of Iba-1 fluorescence intensity revealed significant temporal changes in microglial activation across different age groups. A marked decrease in Iba-1 signal intensity was observed when comparing the following time points: 6 to 12 months, 9 to 12 months, 15 to 17 months, and 15 to 20 months (Figure S3). These reductions suggest a transient downregulation of microglial activation during these intervals.

Conversely, a significant increase in Iba-1 intensity was detected in comparisons between 6 and 15 months, 6 and 17 months, 9 and 15 months, 9 and 17 months, as well as between 12 and 15, 17, and 20 months (Figure S3). These findings indicate a reactivation or upregulation of microglial response at later stages.

**Inner Plexiform Layer (IPL)**

Analysis of Iba-1 fluorescence intensity revealed dynamic changes across different age comparisons. A notable reduction in signal intensity was found between 6 and 9 months, 6 and 12 months, as well as between 15 and 17 months and 15 and 20 months, indicating a significant decrease in microglial activation in these intervals (Figure S3).

In contrast, a significant increase in Iba-1 intensity was observed when comparing 6 to 15 months, 6 to 17 months, and 6 to 20 months. Similarly, comparisons between 9 and 15, 17, and 20 months all showed highly significant increases. Further increases were also evident between 12 and 15, 17, and 20 months, suggesting an increase in microglial activation at later stages (Figure S3).


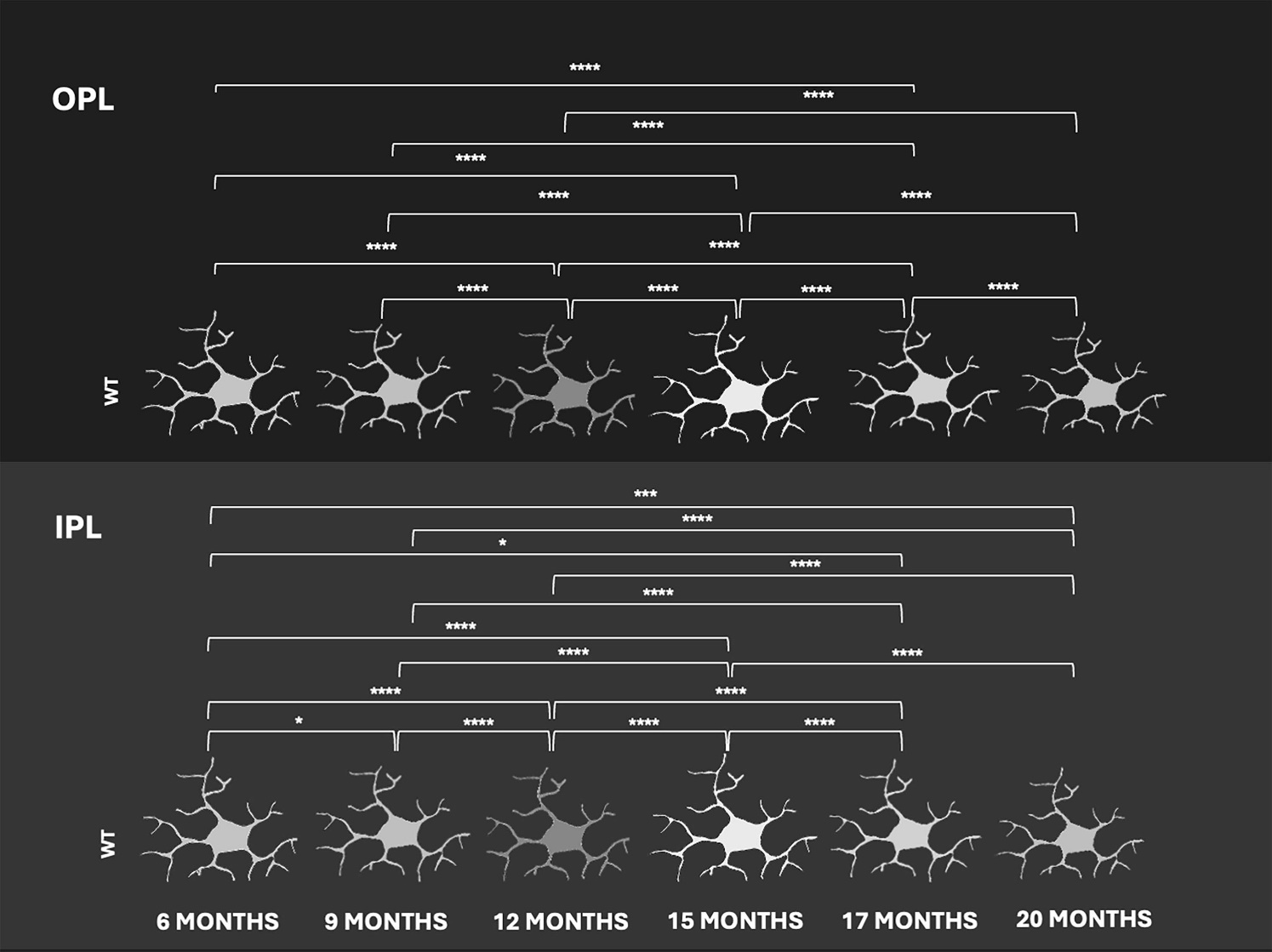


Figure S**3**. Representative schematic comparison of fluorescence intensity of Iba-1 + cells in the outer plexiform layer (OPL) and inner plexiform layer (IPL) of WT mice at 6, 9, 12, 15, 17, and 20 months of age. Whiter tones represent higher fluorescence intensity of Iba-1 staining, while darker gray tones indicate lower intensity, based on a grayscale range from 0 to 250. Statistical significance is shown as p < 0.05 (*), p < 0.01 (**), p < 0.001 (***), and p < 0.0001 (****)

# Supplementary Tables

| **Table S1**. P-values and from the analysis of the **number of Iba1⁺ cells** in the WT and APP^NL-F/NL-F^ models in the **OS layer** | | | | | | | |
| --- | --- | --- | --- | --- | --- | --- | --- |
| **Months** | **6** | **9** | **12** | **15** | **17** | **20** | **WT** |
| **6** |  | 0.9132 | 0.9889 | **0.0478*** | 0.0597 | **<0.0001****** | **6** |
| **9** | 0.997 |  | 0.999 | 0.425 | 0.477 | **<0.0001****** | **9** |
| **12** | 0.218 | 0.075 |  | 0.217 | 0.254 | **<0.0001****** | **12** |
| **15** | **0.0245*** | **0.0054**** | 0.956 |  | >0.9999 | **<0.0001****** | **15** |
| **17** | 0.692 | 0.391 | 0.969 | 0.566 |  | <0.0001**** | **17** |
| **20** | **<0.0001****** | **<0.0001****** | 0.096 | 0.495 | **0.0093**** |  | **20** |
| **APP^NL-F/NL-F^** | **6** | **9** | **12** | **15** | **17** | **20** | **Months** |
| One-way ANOVA with Tukey correction for multiple comparisons.  *p<0.05; **p<0.01; ***p<0.001 and ****<0.0001. | | | | | | | |

| **Table S2.** P-values from the analysis of the **number of Iba1⁺ cells** in the WT and APP^NL-F/NL-F^ models in the **OPL layer** | | | | | | | |
| --- | --- | --- | --- | --- | --- | --- | --- |
| **Months** | **6** | **9** | **12** | **15** | **17** | **20** | **WT** |
| **6** |  | 0.176 | 0.051 | >0.9999 | 0.976 | **<0.0001****** | **6** |
| **9** | 0.111 |  | 0.999 | 0.109 | **0.0281*** | **<0.0001****** | **9** |
| **12** | 0.120 | **<0.0001****** |  | **0.027*** | **0.005**** | **<0.0001****** | **12** |
| **15** | >0.9999 | 0.086 | 0.151 |  | 0.995 | **<0.0001****** | **15** |
| **17** | **0.0021**** | **<0.0001****** | 0.783 | **0.003**** |  | 0.0003*** | **17** |
| **20** | 0.986 | **0.0175*** | 0.427 | 0.994 | **0.0205*** |  | **20** |
| **APP^NL-F/NL-F^** | **6** | **9** | **12** | **15** | **17** | **20** | **Months** |
| One-way ANOVA with Tukey correction for multiple comparisons.  *p<0.05; **p<0.01; ***p<0.001 and ****<0.0001. | | | | | | | |

| **Table S3.** P-values from the analysis of the **number of Iba1⁺ cells** in the WT and APP^NL-F/NL-F^ models in the **IPL layer** | | | | | | | |
| --- | --- | --- | --- | --- | --- | --- | --- |
| **Months** | **6** | **9** | **12** | **15** | **17** | **20** | **WT** |
| **6** |  | 0.980 | **0.001***** | **0.0105*** | **0.0005***** | **<0.0001****** | **6** |
| **9** | 0.342 |  | **0.0001***** | 0.126 | **0.015*** | **<0.0001****** | **9** |
| **12** | **0.046*** | **<0.0001****** |  | **<0.0001****** | **<0.0001****** | **<0.0001****** | **12** |
| **15** | **0.008**** | **<0.0001****** | 0.994 |  | 0.968 | **<0.0001****** | **15** |
| **17** | **0.0102*** | **<0.0001****** | 0.997 | >0.9999 |  | **<0.0001****** | **17** |
| **20** | 0.999 | 0.539 | **0.018*** | **0.0026**** | **0.003**** |  | **20** |
| **APP^NL-F/NL-F^** | **6** | **9** | **12** | **15** | **17** | **20** | **Months** |
| One-way ANOVA with Tukey correction for multiple comparisons.  *p<0.05; **p<0.01; ***p<0.001 and ****<0.0001. | | | | | | | |

| **Table S4**. P-values from the analysis of the **number of Iba1⁺ cells** in the WT and APP^NL-F/NL-F^ models in the **NFL-GCL layer** | | | | | | | |
| --- | --- | --- | --- | --- | --- | --- | --- |
| **Months** | **6** | **9** | **12** | **15** | **17** | **20** | **WT** |
| **6** |  | 0.0003*** | 0.0198* | **<0.0001****** | **<0.0001****** | **<0.0001****** | **6** |
| **9** | 0.595 |  | 0.857 | 0.999 | 0.992 | **<0.0001****** | **9** |
| **12** | **<0.0001****** | **<0.0001****** |  | 0.695 | 0.501 | **<0.0001****** | **12** |
| **15** | **<0.0001****** | **<0.0001****** | 0.981 |  | 1.000 | **<0.0001****** | **15** |
| **17** | **<0.0001****** | **<0.0001****** | 0.759 | 0.988 |  | **<0.0001****** | **17** |
| **20** | **<0.0001****** | **<0.0001****** | **0.0221*** | 0.147 | 0.470 |  | **20** |
| **APP^NL-F/NL-F^** | **6** | **9** | **12** | **15** | **17** | **20** | **Months** |
| One-way ANOVA with Tukey correction for multiple comparisons.  *p<0.05; ****<0.0001. | | | | | | | |

| Table S5. Quantification of the total number of Iba1⁺ cells by layer in the WT group vs. APP^NL-F/NL-F^ at the different study time points | | | | |  |
| --- | --- | --- | --- | --- | --- |
|  |  |  |  |  |  |
| **OS** | | | | |  |
|  | **WT** | **APP^NL-F/NL-F^** |  |  |  |
| **Months** | **Median (IQR)** | **Median (IQR)** | **p-value** | **% difference** |  |
| 6 | 0(0-1) | 1(0-1.75) | **0.0036 **** | ↑122.73 |  |
| 9 | 1(0-2) | 0(0-1) | 0.117 | ↓19.17 |  |
| 12 | 1(0-2) | 1(0-4) | **0.024*** | ↑170.54 |  |
| 15 | 1(0-2) | 2(1-5.5) | **0.019*** | ↑30.66 |  |
| 17 | 1(0-4) | 1(0-3) | 0.894 | ↓8.78 |  |
| 20 | 2.5(0-8) | 2(0-6.75) | 0.788 | ↓21.69 |  |
| **OPL** | | | | |  |
|  | **WT** | **APP^NL-F/NL-F^** |  |  |  |
| **Months** | **Median (IQR)** | **Median (IQR)** | **p-value** | **% difference** |  |
| 6 | 18.5(16-21) | 17(14-19) | **0.0001 ***** | ↓12.70 |  |
| 9 | 17(15-20) | 14(11-18) | **<0.0001****** | ↓16.20 |  |
| 12 | 17(15-19) | 17(14.25-22) | 0.193 | ↑9.54 |  |
| 15 | 19(17-21.75) | 17(14-19) | **0.0002***** | ↓12.96 |  |
| 17 | 19(17-23) | 19(16-22) | 0.8268 | ↑1.07 |  |
| 20 | 22(19-26) | 17(12-22) | **<0.0001 ****** | ↓23.78 |  |
| **IPL** | | | | |  |
|  | **WT** | **APP^NL-F/NL-F^** |  |  |  |
| **Months** | **Median (IQR)** | **Median (IQR)** | **p-value** | **% difference** |  |
| 6 | 15(12.25-16.75) | 15(13-17) | 0.725 | ↓0.4 |  |
| 9 | 15(13-17) | 12.5(11-15.75) | **0.0006 ***** | ↓13.92 |  |
| 12 | 12(10-15) | 17(13-20) | **<0.0001 ****** | ↑39.46 |  |
| 15 | 17(15-18.75) | 17(15.25-19.75) | 0.242 | ↑3.06 |  |
| 17 | 17.5(15-20) | 18(14-20) | 0.682 | ↓0.22 |  |
| 20 | 20(18-23) | 14(8-20) | **<0.0001****** | ↓29.60 |  |
| **RNFL-GCL** | | | | |  |
|  | **WT** | **APP^NL-F/NL-F^** |  |  |  |
| **Months** | **Median (IQR)** | **Median (IQR)** | **p-value** | **% difference** |  |
| 6 | 5(3-7) | 5(2.25-7) | 0.496 | ↓5.71 |  |
| 9 | 8(5.25-9.75) | 3.5(2-6) | **<0.0001****** | ↓48.72 |  |
| 12 | 7(4-9) | 10(8-12) | **<0.0001****** | ↑40.43 |  |
| 15 | 8(6-10) | 9(7-11) | **0.0050**** | ↑20.15 |  |
| 17 | 8(6-10) | 9(7-11) | 0.084 | ↑12.85 |  |
| 20 | 10(8-13) | 7(4.25-10) | **0.0001****** | ↓26.32 |  |
| **Median (Interquartile Range)** of the total number of Iba1⁺ cells in the control group and in the APP^NL-F/NL-F^ model across all analyzed time points and by layers. Mann–Whitney test *p < 0.05; **p < 0.01; ***p < 0.001. ****p < 0.0001. | | | | |  |

| Table S6.P-values from the analysis of the soma size of Iba1+ cells from the WT and APP^NL-F/NL-F^ model in the OPL layer | | | | | | | |
| --- | --- | --- | --- | --- | --- | --- | --- |
| **Months** | **6** | **9** | **12** | **15** | **17** | **20** | **WT** |
| **6** |  | 0.164 | **<0.0001****** | 0.265 | 0.567 | **<0.0001****** | **6** |
| **9** | **<0.0001****** |  | **<0.0001****** | 0.999 | **0.0013 **** | **0.0037**** | **9** |
| **12** | **<0.0001****** | **<0.0001****** |  | **<0.0001****** | **<0.0001****** | **0.001**** | **12** |
| **15** | 0.833 | **<0.0001****** | **<0.0001****** |  | **0.0021**** | **0.0002***** | **15** |
| **17** | **<0.0001****** | **<0.0001****** | **<0.0001****** | **<0.0001****** |  | **<0.0001****** | **17** |
| **20** | **<0.0001****** | **<0.0001****** | **<0.0001****** | **<0.0001****** | 0.157 |  | **20** |
| **APP ^NL-F/NL-F^** | **6** | **9** | **12** | **15** | **17** | **20** | **Months** |
| One-way ANOVA with Tukey correction for multiple comparisons.  **p<0.01 and ****<0.0001. | | | | | | | |

| Table S7.P-values from the analysis of the soma size of Iba1+ cells from the WT and APP^NL-F/NL-F^ model in the IPL layer | | | | | | | |
| --- | --- | --- | --- | --- | --- | --- | --- |
| **Months** | **6** | **9** | **12** | **15** | **17** | **20** | **WT** |
| **6** |  | 0.310 | **<0.0001****** | **0.001***** | 0.969 | **0.032*** | **6** |
| **9** | **<0.0001****** |  | **<0.0001****** | 0.536 | **0.0483*** | 0.986 | **9** |
| **12** | **0.046*** | **<0.0001****** |  | **<0.0001****** | **<0.0001****** | **<0.0001****** | **12** |
| **15** | 0.422 | **<0.0001****** | **<0.0001****** |  | **<0.0001****** | 0.838 | **15** |
| **17** | **<0.0001****** | 0.079 | **<0.0001****** | **<0.0001****** |  | **0.001**** | **17** |
| **20** | **<0.0001****** | 0.926 | **<0.0001****** | **<0.0001****** | **0.002**** |  | **20** |
| **APP ^NL-F/NL-F^** | **6** | **9** | **12** | **15** | **17** | **20** | **Months** |
| One-way ANOVA with Tukey correction for multiple comparisons.  *p<0.05; **p<0.01; ***p<0.001 and ****<0.0001. | | | | | | | |

| Table 8. Soma size of Iba1⁺ cells by layer in the WT group vs. APP^NL-F/NL-F^ at the different study time points | | | | |  |
| --- | --- | --- | --- | --- | --- |
|  |  |  |  |  |  |
| **OPL** | | | | |  |
|  | **WT** | **APP^NL-F/NL-F^** |  |  |  |
| **Months** | **Median (IQR)** | **Median (IQR)** | **p-value** | **% difference** |  |
| 6 | 36.34(27.61-47) | 39.87(28.79-56.16) | **<0.0001 ****** | ↑19.36 |  |
| 9 | 38.29(29.7-49.18) | 49.37(35.92-69.29) | **<0.0001****** | ↑34.86 |  |
| 12 | 49.37(35.92-69.29) | 34.36(27.18-43.01) | **<0.0001****** | ↓32.91 |  |
| 15 | 37.36(28.91-48.18) | 40.82(31.07-54.94) | **<0.0001****** | ↑11.57 |  |
| 17 | 34.72(28.05-44.45) | 53.4(40.8-72.66) | **<0.0001****** | ↑61.32 |  |
| 20 | 38.63(29.22-52.56) | 58.43(42.71-79.69) | **<0.0001****** | ↑45.48 |  |
| **IPL** | | | | |  |
|  | **WT** | **APP^NL-F/NL-F^** |  |  |  |
| **Months** | **Median (IQR)** | **Median (IQR)** | **p-value** | **% difference** |  |
| 6 | 34.51(25.61-45.79) | 35.15(25.34-47.86) | 0.198 | ↑5.8 |  |
| 9 | 36.88(27.25-47.74) | 47.5(32.28-73.8) | **<0.0001****** | ↑54.72 |  |
| 12 | 40.62(31.16-53.55) | 32.61(24.68-42.28) | **<0.0001****** | ↓20.17 |  |
| 15 | 38.07(28.19-49.1) | 37.65(27.95-49.79) | 0.8846 | ↑3.13 |  |
| 17 | 35.28(26.42-44.69) | 51.37(41.28-65.49) | **<0.0001****** | ↑55.75 |  |
| 20 | 36.1(26.87-47.4) | 56.98(39.39-81.04) | **<0.0001****** | ↑70.25 |  |
| **Median (Interquartile Range)** of the soma size of Iba1⁺ cells in the control group and in the APP^NL-F/NL-F^ model across all analyzed time points and by layers. Mann–Whitney test ****p < 0.0001. | | | | |  |

| Table S9. P-values from the analysis of the arborization area of Iba1⁺ cells in the WT and APP^NL-F/NL-F^ models in the OPL layer | | | | | | | |
| --- | --- | --- | --- | --- | --- | --- | --- |
| **Months** | **6** | **9** | **12** | **15** | **17** | **20** | **WT** |
| **6** |  | **<0.0001****** | 0.896 | **0.0133*** | **0.017*** | **<0.0001****** | **6** |
| **9** | **<0.0001***** |  | **0.0002***** | **<0.0001****** | 0.141 | **<0.0001****** | **9** |
| **12** | **0.002**** | **<0.0001***** |  | **0.0003***** | 0.296 | **<0.0001****** | **12** |
| **15** | 0.062 | **<0.0001***** | **<0.0001***** |  | **<0.0001****** | **<0.0001****** | **15** |
| **17** | **0.001**** | **<0.0001***** | >0.999 | **<0.0001***** |  | **<0.0001****** | **17** |
| **20** | **<0.0001****** | **<0.0001***** | **<0.0001***** | **<0.0001***** | **<0.0001***** |  | **20** |
| **APP^NL-F/NL-F^** | **6** | **9** | **12** | **15** | **17** | **20** | **Months** |
| One-way ANOVA with Tukey correction for multiple comparisons.  *p<0.05; **p<0.01; ***p<0.001 and ****<0.0001. | | | | | | | |

| Table S10. P-values from the analysis of the arborization area of Iba1⁺ cells in the WT and APP^NL-F/NL-F^ models in the IPL layer | | | | | | | |
| --- | --- | --- | --- | --- | --- | --- | --- |
| **Months** | **6** | **9** | **12** | **15** | **17** | **20** | **WT** |
| **6** |  | >0.9999 | >0.9999 | **0.003**** | 0.160 | **<0.0001****** | **6** |
| **9** | **<0.0001****** |  | 0.999 | **0.001**** | 0.211 | **<0.0001****** | **9** |
| **12** | **0.002**** | **<0.0001****** |  | **0.006**** | 0.147 | **<0.0001****** | **12** |
| **15** | **<0.0001****** | **<0.0001****** | 0.075 |  | **<0.0001****** | **0.005**** | **15** |
| **17** | **<0.0001****** | **<0.0001****** | 0.083 | **<0.0001****** |  | **<0.0001****** | **17** |
| **20** | **<0.0001****** | 0.891 | **<0.0001****** | **<0.0001****** | **<0.0001****** |  | **20** |
| **APP NL-F/NL-F** | **6** | **9** | **12** | **15** | **17** | **20** | **Months** |
| One-way ANOVA with Tukey correction for multiple comparisons.  **p<0.01; and ****<0.0001 | | | | | | | |

| Table S11. Arborization area of Iba1⁺ cells by layer in the WT group vs. APP^NL-F/NL-F^ at the different study time points | | | | |
| --- | --- | --- | --- | --- |
|  |  |  |  |  |
| **OPL** | | | | |
|  | **WT** | **APP^NL-F/NL-F^** |  |  |
| **Months** | **Median (IQR)** | **Median (IQR)** | **p-value** | **% difference** |
| 6 | 4100(2809-5350) | 4227(2951-5606) | 0.16 | ↑3.61 |
| 9 | 4693(3195-5891) | 2467(1094-4605) | **<0.0001****** | ↓34.77 |
| 12 | 4169(2936-5635) | 3798(2264-5294) | **<0.0001****** | ↓7.55 |
| 15 | 3780(2565-5058) | 4544(3127-6062) | **<0.0001****** | ↑18.85 |
| 17 | 4324(3270-5580) | 3805(2330-5351) | **<0.0001****** | ↓10.95 |
| 20 | 3310(2258-4433) | 3422(1900-4732) | 0.6182 | ↑0.75 |
| **IPL** | | | | |
|  | **WT** | **APP^NL-F/NL-F^** |  |  |
| **Months** | **Median (IQR)** | **Median (IQR)** | **p-value** | **% difference** |
| 6 | 3386(1961-4941) | 3537(2320-5063) | **0.0280*** | ↑5.19 |
| 9 | 3404(2016-4934) | 2059(977.8-3906) | **<0.0001****** | ↓24.90 |
| 12 | 3252(1896-4979) | 3153(1859-4567) | 0.1509 | ↓5.26 |
| 15 | 2992(1771-4363) | 3450(2256-4930) | **<0.0001****** | ↑13.59 |
| 17 | 3625(2307-5123) | 2793(1548-4400) | **<0.0001****** | ↓17.51 |
| 20 | 2669(1700-3810) | 2188(1025-3588) | **<0.0001****** | ↓9.21 |
| **Median (Interquartile Range)** of the arborization area of Iba1⁺ cells in the control group and in the APP^NL-F/NL-F^ model across all analyzed time points and by layers. Mann–Whitney test ****p < 0.0001. | | | | |

| **Table S12.** P-values from the analysis of the **skeletonization area** of Iba1⁺ cells in the WT and APP^NL-F/NL-F^ models in the **OPL layer** | | | | | | | |
| --- | --- | --- | --- | --- | --- | --- | --- |
| **Months** | **6** | **9** | **12** | **15** | **17** | **20** | **WT** |
| **6** |  | **<0.0001****** | 0.311 | **0.006**** | >0.9999 | **<0.0001****** | **6** |
| **9** | **<0.0001****** |  | **<0.0001****** | **<0.0001****** | **<0.0001****** | **<0.0001****** | **9** |
| **12** | 0.995 | **<0.0001****** |  | 0.656 | 0.321 | 0.181 | **12** |
| **15** | **0.001****** | **<0.0001****** | **0.003**** |  | **0.006**** | 0.989 | **15** |
| **17** | **<0.0001****** | **<0.0001****** | **<0.0001****** | **<0.0001****** |  | **<0.0001****** | **17** |
| **20** | **<0.0001****** | **<0.0001****** | **<0.0001****** | **<0.0001****** | **0.001***** |  | **20** |
| **APP^NL-F/NL-F^** | **6** | **9** | **12** | **15** | **17** | **20** | **Months** |
| One-way ANOVA with Tukey correction for multiple comparisons.  **p<0.01; ***p<0.001 and ****<0.0001. | | | | | | | |

| Table S13. P-values from the analysis of the skeletonization area of Iba1⁺ cells in the WT and APP^NL-F/NL-F^ models in the IPL layer | | | | | | | |
| --- | --- | --- | --- | --- | --- | --- | --- |
| **Months** | **6** | **9** | **12** | **15** | **17** | **20** | **WT** |
| **6** |  | 0.370 | 0.995 | **0.005**** | 0.999 | **0.001***** | **6** |
| **9** | **0.023*** |  | 0.156 | 0.542 | 0.570 | 0.278 | **9** |
| **12** | 0.992 | 0.087 |  | **0.001**** | 0.941 | **<0.0001****** | **12** |
| **15** | 0.889 | **0.0001**** | 0.494 |  | **0.011*** | **0.999** | **15** |
| **17** | **<0.0001****** | **<0.0001****** | **<0.0001****** | **<0.0001****** |  | **0.001**** | **17** |
| **20** | **<0.0001****** | **<0.0001****** | **<0.0001****** | **<0.0001****** | 0.067 |  | **20** |

One-way ANOVA with Tukey correction for multiple comparisons.

*p<0.05; **p<0.01; ***p<0.001 and ****<0.0001.

| Table S14. Skeletonization area of Iba1⁺ cells by layer in the WT group vs. APP^NL-F/NL-F^ at the different study time points | | | | |  |
| --- | --- | --- | --- | --- | --- |
|  |  |  |  |  |  |
| **OPL** | | | | |  |
|  | **WT** | **APP ^NL-F/NL-F^** |  |  |  |
| **Months** | **Median (IQR)** | **Median (IQR)** | **p-value** | **% difference** |  |
| 6 | 1037(675.2-1530) | 1042(646.4-1475) | 0.631 | ↑1.4 |  |
| 9 | 1192(828.3-1678) | 812.9(437.7-1375) | **<0.0001****** | ↓23.35 |  |
| 12 | 980.1(631-1467) | 1047(659.9-1502) | 0.051 | ↑8.68 |  |
| 15 | 961.2(614.3-1355) | 1176(740.2-1674) | **<0.0001****** | ↑25.00 |  |
| 17 | 1058(668.9-1515) | 564.8(365.4-853.4) | **<0.0001****** | ↓40.14 |  |
| 20 | 932(608.2-1332) | 617.3(377.7-984.9) | **<0.0001****** | ↓21.98 |  |
| **IPL** | | | | |  |
|  | **WT** | **APP^NL-F/NL-F^** |  |  |  |
| **Months** | **Median (IQR)** | **Median (IQR)** | **p-value** | **% difference** |  |
| 6 | 964.4(556.2-1462) | 943.5(545.3-1464) | 0.9049 | ↓3.12 |  |
| 9 | 932.6(552.3-1372) | 748.9(424-1254) | **<0.0001****** | ↓6.60 |  |
| 12 | 950.9(567-1485) | 919.7(551.9-1398) | 0.3283 | ↓6.56 |  |
| 15 | 835.8(519.6-1353) | 986.5(599.8-1458) | **0.0007***** | ↑13.72 |  |
| 17 | 973.7(590.2-1483) | 594.9(367.2-888.9) | **<0.0001****** | ↓38.27 |  |
| 20 | 866.2(526.9-1281) | 591.6(335.1-1071) | **<0.0001****** | ↓22.13 |  |
| **Median (Interquartile Range)** of the skeletonization area of Iba1⁺ cells in the control group and in the APP^NL-F/NL-F^ model. across all analyzed time points and by layers. Mann–Whitney test ***p < 0.001; ****p < 0.0001. | | | | |  |

| **Table S15.** P-values from the analysis of **the Feret´s Diameter Ratio** of Iba1⁺ cells in the WT and APP^NL-F/NL-F^ models in the O**PL layer** | | | | | | | |
| --- | --- | --- | --- | --- | --- | --- | --- |
| **Months** | **6** | **9** | **12** | **15** | **17** | **20** | **WT** |
| **6** |  | 0.939 | 1.000 | 0.950 | 0.982 | >0.9999 | **6** |
| **9** | 0.570 |  | 0.993 | >0.9999 | >0.9999 | 0.970 | **9** |
| **12** | **<0.0001****** | **0.0003***** |  | 0.994 | 0.999 | >0.9999 | **12** |
| **15** | 0.399 | 0.999 | **0.003*** |  | >0.9999 | 0.976 | **15** |
| **17** | **0.041*** | 0.789 | **0.047*** | 0.943 |  | 0.994 | **17** |
| **20** | >0.999 | 0.680 | **<0.0001****** | 0.503 | 0.067 |  | **20** |
| **APP^NL-F/NL-F^** | **6** | **9** | **12** | **15** | **17** | **20** | **Months** |
| One-way ANOVA with Tukey correction for multiple comparisons.  *p<0.05; ***p<0.001 and ****<0.0001. | | | | | | | |

| Table S16. P-values from the analysis of the Feret´s Diameter Ratio of Iba1⁺ cells in the WT and APP^NL-F/NL-F^ models in the IPL layer | | | | | | | |
| --- | --- | --- | --- | --- | --- | --- | --- |
| **Months** | **6** | **9** | **12** | **15** | **17** | **20** | **WT** |
| **6** |  | 0.928 | 0.976 | 0.889 | 0.290 | 0.850 | **6** |
| **9** | 0.120 |  | >0.9999 | >0.9999 | 0.846 | >0.9999 | **9** |
| **12** | **0.003**** | 0.851 |  | 0.999 | 0.824 | 0.999 | **12** |
| **15** | **0.014*** | 0.969 | 0.999 |  | 0.918 | >0.999 | **15** |
| **17** | 0.166 | >0.999 | 0.806 | 0.951 |  | 0.915 | **17** |
| **20** | 0.478 | 0.978 | 0.389 | 0.654 | 0.990 |  | **20** |
| **APP^NL-F/NL-F^** | **6** | **9** | **12** | **15** | **17** | **20** | **Months** |
| One-way ANOVA with Tukey correction for multiple comparisons.  *p<0.05; **p<0.01 | | | | | | | |

| Table S17. Feret´s Diameter Ratio of Iba1⁺ cells by layer in the WT group vs. APP^NL-F/NL-F^ at the different study time points | | | | |  |
| --- | --- | --- | --- | --- | --- |
|  |  |  |  |  |  |
| **OPL** | | | | |  |
|  | **WT** | **APP^NL-F/NL-F^** |  |  |  |
| **Months** | **Median (IQR)** | **Median (IQR)** | **p-value** | **% difference** |  |
| 6 | 1.89(1.39-2.395) | 1.98(1.54-2.47) | 0.1739 | ↑3.59 |  |
| 9 | 1.755(1.398-2.303) | 1.79(1.42-2.33) | 0.6625 | ↑1.52 |  |
| 12 | 1.915(1.398-2.41) | 1.625(1.28-2.045) | **<0.0001****** | ↓13.72 |  |
| 15 | 1.78(1.42-2.388) | 1.875(1.41-2.42) | 0.7251 | ↑0.35 |  |
| 17 | 1.87(1.34-2.43) | 1.77(1.34-2.27) | 0.2072 | ↓3.42 |  |
| 20 | 1.83(1.38-2.53) | 1.95(1.44-2.505) | 0.1677 | ↑3.66 |  |
| **IPL** | | | | |  |
|  | **WT** | **APP^NL-F/NL-F^** |  |  |  |
| **Months** | **Median (IQR)** | **Median (IQR)** | **p-value** | **% difference** |  |
| 6 | 1.75(1.37-2.22) | 1.98(1.47-2.42) | **0.0085**** | ↑9.47 |  |
| 9 | 1.755(1.34-2.473) | 1.77(1.37-2.16) | 0.5433 | ↓2.47 |  |
| 12 | 1.84(1.37-2.33) | 1.635(1.32-2.083) | **0.0145*** | ↓5.96 |  |
| 15 | 1.825(1.383-2.3) | 1.73(1.36-2.155) | 0.1706 | ↓5.74 |  |
| 17 | 1.84(1.44-2.35) | 1.685(1.35-2.215) | **0.0317*** | ↓5.71 |  |
| 20 | 1.82(1.39-2.29) | 1.78(1.39-2.32) | 0.8788 | ↓0.56 |  |
| **Median (Interquartile Range)** of the FDR of Iba1⁺ cells in the control group and in the APP^NL-F/NL-F^ model across all analyzed time points and by layers. Mann–Whitney test *p < 0.05; **p < 0.01; ****p < 0.0001. | | | | |  |

| Table S18. Fluorescence intensity of Iba1⁺ cells by layer in the WT group vs. APP^NL-F/NL-F^ at the different study time points | | | | |  |
| --- | --- | --- | --- | --- | --- |
|  |  |  |  |  |  |
| **OPL** | | | | |  |
|  | **WT** | **APP^NL-F/NL-F^** |  |  |  |
| **Months** | **Mean** ± **SD** | **Mean** ± **SD** | **p-value** | **% difference** |  |
| 6 | 195.6 ± 38.94) | 230.7± 28.86 | **<0.0001 ****** | ↑17.94 |  |
| 9 | 191.4±53.51 | 225.4±34.37 | **<0.0001****** | ↑17.76 |  |
| 12 | 135.5±39.54 | 219.1±50.87 | **<0.0001****** | ↑61.70 |  |
| 15 | 234±30.07 | 221.1±36.33 | **<0.0001****** | ↓5.51 |  |
| 17 | 209.9±28.67 | 222.2±36.49 | **<0.0001****** | ↑5.86 |  |
| 20 | 191.2±47.65 | 155.7±45.09 | **<0.0001****** | ↓18.57 |  |
| **IPL** | | | | |  |
|  | **WT** | **APP^NL-F/NL-F^** |  |  |  |
| **Months** | **Mean** ± **(SD)** | **Mean** ± **(SD)** | **p-value** | **% difference** |  |
| 6 | 196.1± 49.07 | 229.7±29.9 | **<0.0001****** | ↑17.13 |  |
| 9 | 185.2±45.24 | 216.9±37.29 | **<0.0001****** | ↑17.13 |  |
| 12 | 156.8±42.8 | 206.1±45.26 | **<0.0001****** | ↑31.44 |  |
| 15 | 231.7±32.41 | 222.5±31.52 | **<0.0001****** | ↓3.97 |  |
| 17 | 207.9±38.96 | 215±40.25 | **<0.01**** | ↑3.42 |  |
| 20 | 210.7±36.01 | 168.6 ±38.41 | **<0.0001****** | ↓19.98 |  |
| **Mean** ± (Std. Deviation) of Fluorescence intensity of Iba1⁺ cells in the control group and in the APP^NL-F/NL-F^ model across all analyzed time points and by layers. Mann–Whitney test **p < 0.01; ****p < 0.0001. | | | | |  |
